# Identifying behaviour change techniques within precision health interventions that use continuous glucose monitoring: a secondary analysis of a scoping review

**DOI:** 10.1186/s12966-025-01833-5

**Published:** 2025-11-06

**Authors:** Lauren Connell Bohlen, Jacob Crawshaw, Michelle R. Jospe, Kelli M. Richardson, Kristin J. Konnyu, Susan M. Schembre

**Affiliations:** 1 Center for Health Promotion and Health Equity, Department of Behavioural and Social Sciences, Brown University School of Public Health, Providence, RI USA; 2 Methodological and Implementation Research Program, Ottawa Hospital Research Institute, Ottawa, ON Canada; 3 Department of Oncology, Lombardi Comprehensive Cancer Center, Georgetown University, 2115 Wisconsin Avenue NW Suite 300, Washington, DC 20007 USA; 4 Center for Evidence Synthesis and Health, Department of Health Services Policy and Practice, Brown University School of Public Health, Providence, RI USA; 5 Health Services Research Unit, University of Aberdeen, Aberdeen, Scotland

**Keywords:** Continuous glucose monitoring, Behaviour change, Glycaemic control, Glycated haemoglobin, Precision medicine, Precision health, Digital health, Behavioural theory, Behaviour change technique

## Abstract

**Background:**

Continuous glucose monitoring (CGM) is increasingly being used within precision health interventions to motivate behaviour change. However, there is considerable variability and complexity in the design of behaviour change interventions that incorporate CGM-based biofeedback, making it challenging to disentangle the intervention components that are driving intervention effects. The objective of this review is to identify the behaviour change techniques and mechanisms of action commonly implemented alongside CGM-based biofeedback.

**Methods:**

We conducted secondary analyses of a scoping review to identify health behaviour interventions (RCTs) that provided CGM-based biofeedback to promote behaviour change in adults. Two researchers applied the 93-item Behaviour Change Techniques (BCT) Taxonomy (v1) to independently code intervention content in all trial arms (i.e., intervention and comparison arms) dependent upon their targeted behaviour of CGM use, glucometer use, diet, physical activity, or medication adherence. BCTs were analysed individually and according to their corresponding category. We performed univariate linear regression analyses to examine whether the presence of individual BCTs and target behaviours influenced pre-post changes in HbA1c within CGM-based intervention arms.

**Results:**

Thirty-one RCTs comprising 35 intervention arms and 29 comparison arms were included. Theory was reported in 4 studies (13%), most commonly Self-Efficacy Theory. Mechanisms of action (MoAs) were specified in 5 studies (16%), typically targeting beliefs about capabilities. We identified 40 (of 93 possible) unique BCTs, with intervention arms employing an average of 7.1 BCTs (SD: 4.8) compared to 5.3 BCTs (SD: 4.3) in comparison arms. The most frequently implemented BCT categories in CGM-based biofeedback interventions were ‘*Feedback and monitoring*’ (*n* = 35/35, 100%), ‘*Shaping knowledg*e’ (*n* = 28/35, 80%), and ‘*Social support*’ (*n* = 22/35, 63%). Commonly used BCTs supporting CGM use and promoting dietary and physical activity changes included ‘*Biofeedback*’ (*n* = 35/35; 100%), ‘*Instruction on how to perform the behaviour*’ (*n* = 19/35; 54%), and ‘*Credible source*’ (*n* = 14/35; 40%). Univariate linear regressions did not identify any individual BCTs or targeted behaviours that significantly moderated HbA1c outcomes.

**Conclusions:**

RCTs using CGM to change behaviour in adult populations include a range of BCTs, focusing predominantly on BCTs that support the implementation of CGM itself. Future research should examine whether BCTs operate through distinct MoAs when supporting CGM uptake and use versus when promoting broader health behaviour change in conjunction with CGM-based biofeedback.

**Supplementary Information:**

The online version contains supplementary material available at 10.1186/s12966-025-01833-5.

## Background

Many chronic diseases, including diabetes and cardiovascular disease, can be prevented or treated through behavioural interventions targeting diet and activity [1]. However, the effectiveness of conventional one-size-fits all intervention approaches often falls short, as individual responses to interventions can vary widely. In contrast, precision health interventions leverage individual genetic, behavioural, and environmental data to deliver personalized strategies that enhance intervention effectiveness, improve adherence, and optimize health outcomes [2]. Several meta-analyses have shown the superiority of this personalized approach compared to standardized interventions [3–5]. More modern day behaviour change interventions are being designed in accordance with the tenets of precision health [6, 7]. 

One behaviour change technique that has been increasingly used within the realm of precision health interventions is biofeedback. Biofeedback involves providing individuals with personalized “feedback about the body’s physiological or biochemical state using an external monitoring device as part of a behaviour change strategy” [8, 9]. An example of this is providing an individual with their cholesterol levels to motivate dietary change. Despite the burgeoning implementation of biofeedback to motivate health behaviour change [8], little is known about the mechanisms of action (MoAs) by which biofeedback facilitates behaviour change [10]. To address this, our research team conducted two scoping reviews and one systematic review and meta-analysis. Our first scoping review of 767 randomised controlled trials (RCTs) [8] aimed to describe the domains of research where biofeedback is being used, as well as the methodological characteristics of biofeedback in behaviour change research. While study characteristics and implementation of biofeedback varied widely in the scoping review, results revealed biofeedback’s predominant application within diabetes research, with self-monitored glucose levels being one of the most frequently leveraged biological markers of health [8]. 

The predominance of glucose-based biofeedback may be reasonably attributed to the longstanding history and ongoing evolution of glucose monitoring as a tool to improve diabetes-related treatment and health outcomes [11]. There are fewer barriers to obtaining and utilizing glucose-based biofeedback. Glucose monitoring has become less invasive, transitioning from glucometers, which employ a finger prick method and offer biofeedback on a single glucose value, to continuous glucose monitors (CGMs), which are minimally invasive wearable devices that deliver continuous biofeedback on glucose trends that are visible to users on their smartphones for at least 10 days. These data and data visualizations can provide insights into how health behaviours, such as diet and physical activity (PA), affect their glucose levels, empowering individuals (with and without diabetes) to make informed behaviour changes to improve related health outcomes (e.g., average glucose levels, time-in-range) [12]. We conducted a systematic review and meta-analysis which showed that behaviour change interventions incorporating CGM-based biofeedback among people with and without diabetes produced a 0.3 unit (%) greater reduction in glycated haemoglobin (HbA1c) and a 7% increase in time-in-range (mins) compared to controls without CGM, demonstrating CGM’s efficacy as a behaviour change tool, regardless of diabetes status [13]. Despite the evidence that this innovative, digital approach to behaviour change has gained traction in multiple health-focused sectors, including healthcare [14], digital startup companies, and in research [15], the methods by which CGMs are employed in health behaviour change interventions had not yet been characterized.

In an effort to add to the paucity of literature examining the implementation characteristics of studies that incorporate CGM-based biofeedback, our team conducted a second scoping review aimed to characterize the methods of incorporating CGM-based biofeedback into behaviour change interventions [15]. Findings from 31 RCTs revealed considerable heterogeneity in the protocols used to deliver CGM-based biofeedback, such that all studies employed complex multi-component interventions, frequently pairing CGM with prospective or retrospective guidance on interpreting CGM data, health-related education, and diet, PA, or medication tracking [15]. This variability and complexity highlights the lack of consensus on best practices for designing biofeedback interventions. Continuing this line of research will help to establish evidence-based guidance for designing precision health interventions that incorporate CGM-based biofeedback.

Here, we have taken what we believe is the next most important step towards intervention optimization when using CGM-based biofeedback as part of a behaviour change strategy. To identify the most effective and replicable approaches for applying CGM-based biofeedback within behaviour change interventions, we believe it is essential to investigate the specific behaviour change techniques (BCTs) that accompany CGM-based biofeedback to better understand why the use of CGMs produces improvement in diabetes-related health outcomes that are superior to non-CGM based interventions. BCTs are defined as “observable, replicable, and irreducible components of an [behaviour change] intervention” [9]. Over the past 20 years, significant efforts have been made to develop structured taxonomies of BCTs, with the most extensive and widely recognized being the Behaviour Change Technique Taxonomy version 1 (BCTTv1) [9]. The BCTTv1 categorizes 93 distinct BCTs grouped into 16 categories, with each BCT accompanied by a label, definition, and practical example of the BCT in-action. The BCTTv1 serves as a standardized framework for identifying and categorizing BCTs within behaviour change interventions, helping to pinpoint the ‘active ingredients’ of an intervention. The taxonomy has been used extensively to build interventions from the ground up [16] and has also demonstrated value in supporting evidence synthesis to help unpack the content of existing behaviour change interventions [17], which are generally poorly reported across all study arms [18]. While BCTs can be used independently, they often work synergistically, making their identification critical for understanding the theoretical basis for how and why an intervention works. Continuous advancements, such as the development of the Behaviour Change Technique Ontology [19], have improved the precision of BCT coding, enhancing researcher’s ability to evaluate the active intervention components. However, the use of behaviour change theory to guide CGM-based biofeedback interventions remains underused, leaving uncertainty about how biofeedback functions within the broader intervention [8, 15, 20]. Despite the demonstrated efficacy of CGM-based biofeedback interventions, little is known about the BCTs and MoAs used alongside CGM. This study aimed to address this gap by using established coding frameworks to identify and specify the behaviour change content (i.e., BCTTv1) [9] and MoAs [10, 21] within RCTs of interventions using CGM-based biofeedback. We also examined whether individual BCTs were associated with changes in glycaemic outcomes. Given the limited number of studies and the variability in intervention design, this study represents a first step toward understanding how CGM interacts with other behavioural strategies in the context of precision health interventions.

## Methods

### Study design and selection

This preregistered study (PROSPERO - CRD42023398390) [22] reports a secondary analysis of the published scoping review introduced above [15]. The scoping review adhered to The Preferred Reporting Items for Systematic Reviews and Meta-Analyses Extension for Scoping Reviews (PRISMA-ScR) checklist [23] and was registered on Open Science Framework Registries (OSF.IO/SJREA) [24].

Details of the search strategy and eligibility criteria for the included CGM studies are reported elsewhere [15]. Briefly, we sought RCTs targeting adults that evaluated interventions using CGM-based biofeedback for behaviour change in at least one study arm. CGM-based biological feedback could include unblinded CGM-based feedback whereby participants have access to their glucose data in real-time, or blinded CGM-based feedback whereby participants’ glucose data was collected and then reviewed by a provider with the participant at a later date. We extracted bibliographical details, participant characteristics, outcomes, targeted behaviours, intervention details, CGM use, and the use of behaviour change theory. Articles were included up to January 2024.

### Data extraction

This study expands on our previous work [15] such that we used the BCTTv1 to specify the behaviour change content within all study arms, as well as any MoAs that were explicitly reported in the included studies based on established coding guidelines for extracting this information from published intervention reports [22]. We used all relevant published source material (i.e. manuscripts, supplementary materials, and study protocols) to specify BCTs used within each study. Two researchers (LCB and JC), trained and experienced in using the BCTTv1 [10, 21, 25–27], independently coded the intervention content across all study arms. Inter-rater reliability was not calculated; instead we used a consensus-based approach in which all papers were double-coded and coding was compared to identify discrepancies and to develop coding rules [25]. Discrepancies which could not be resolved by the two primary BCTTv1 coders (LCB, JC), were brought to the larger team for discussion and to make a final decision.

### BCT coding framework

The original published BCTTv1 was used as an initial coding framework. Generic definitions and examples from the BCTTv1 were adapted iteratively during the coding process to reflect our CGM dataset. Our consensus-based approach to coding meant that there was ample opportunity to generate specific coding rules during the data extraction process. For example, we adhered to the guideline to code, “All BCTs…whether they are targeting one or more of the target behaviours or key preparatory behaviours of the intervention, including engagement, or implementation of the intervention.” Other specific BCT coding rules included: (i) Not coding BCTs associated with using glucometers in conjunction with CGM, if this is for calibration purposes only; (ii) If CGM is blinded, and no feedback is provided, do not code ‘*Biofeedback’*, code as ‘*Monitoring of outcome(s) without feedback*’; (iii) Do not code pharmacological support when insulin modifications are part of the CGM strategy. Our entire list of coding rules is reported in Appendix 1. A requirement of the included studies for this analysis was that the intervention arm implemented CGM; thus the BCT ‘*Biofeedback’* was coded in every study.

### Data analysis

Descriptive analyses were conducted to detail the frequency and distribution of BCTs by target behaviour, and by intervention or comparison arm. Fisher’s exact tests were conducted for each BCT category to assess differences in proportions between comparison and intervention arms. To account for multiple comparisons, *p*-values were adjusted using the Benjamini-Hochberg procedure with a significance level set at α = 0.05.

We performed univariate linear regression analyses to examine how individual BCTs and target behaviours influenced pre-post changes in HbA1c within 31 intervention arms with available HbA1c data. For each BCT, a binary indicator variable was created, and separate models were fitted using the lm() function in R (version 2023.06.1; R Core Team, 2023). Each regression estimated the association between BCT presence and pre-post HbA1c change across all CGM-based intervention arms. A two-tailed p-value of < 0.05 was considered statistically significant. Analyses were limited to intervention arms to specifically assess BCTs in the context of CGM-based biofeedback. Including comparison arms (which did not implement CGM) would have confounded the interpretation of how BCTs relate to glycaemic outcomes in CGM-enabled interventions. To ensure sufficient variability for meaningful analysis, we included only BCTs that appeared in 10–90% of studies, following established methodological approaches [28, 29]. The analysis evaluated both the presence or absence of specific BCTs and the inclusion of key target behaviours (dietary intake, PA, medication adherence, and use of glucometers) as predictors of intervention effectiveness, measured by changes in HbA1c. Dietary intake was excluded from the analysis as it was present across all studies, providing no variability for comparison.

## Results

### Study characteristics

Study characteristics are summarized in Appendix 3. Additional details can be found elsewhere [15]. Briefly, 3961 articles were screened for eligibility, and 31 RCTs met our criteria [30–60]. Target populations were predominantly of a clinical nature, including those with type 2 diabetes (*n* = 20/31, 65%), pregestational and gestational diabetes (*n =* 6/31, 19%), type 1 diabetes (*n =* 4/31, 13%), overweight and obesity (*n* = 4/31, 13%), and prediabetes (*n* = 1/31, 3%). In total, there were 35 intervention arms, defined as those which implemented CGM-based biofeedback, and 29 comparison arms, which did not use CGM-based biofeedback.

### Behavioural theory and mechanisms of action (MoAs)

Theory was reported in 4 of 31 (13%) studies, with 6 distinct behaviour change theories being mentioned as underlying the development of the intervention. Most frequently, interventions were grounded in Self-Efficacy Theory (*n* = 3), which assumes that an individual’s belief in their own abilities predicts their behaviour [61]. Other cited theories included Social Cognitive Theory (*n* = 2), Health Belief Model (*n* = 1), Social Learning Theory (*n* = 1), the Integrated Behavioural Model (*n* = 1), and Goals, Reality, Options and Will Model (*n* = 1).

MoAs were reported in 5 of 31 (16%) of studies [21]. In three studies, the intervention was specifically designed to target beliefs about capabilities (i.e., self-efficacy) [30, 32, 45]. One study specified that CGM-based biofeedback was specifically intended to increase motivation to change diet and PA behaviours associated with desirable changes in blood glucose [38]. In another study, the intervention was designed to modify behavioural regulation (i.e., self-regulation) by combining a cluster of BCTs (*‘Information about antecedents*,*’ ‘Behavioural experiments*,*’ ‘Instruction on how to perform the behaviour*,*’ ‘Information about health consequences’*) with CGM-based biofeedback, ultimately to prompt weight loss-related behaviors [50]. 

### Behaviour change techniques

Across all study arms, 40 of 93 possible BCTs were coded at least once. The number of BCTs included in each intervention arm ranged from 2 to 21 (mean: 7.1; SD: 4.8), while BCTs in comparison arms ranged from 0 to 20 (mean: 5.3; SD: 4.3). BCTs could have more than 1 target behaviour. BCTs targeted CGM use in 35 intervention arms (100%) and 6 comparison arms (21%), and targeted glucometer use in 9 intervention arms (26%) and 23 comparison arms (79%). BCTs aimed to promote dietary changes in 33 intervention arms (94%) and 26 comparison arms (90%); PA changes in 26 intervention arms (74%) and 19 comparison arms (66%); and medication adherence in 4 intervention arms (11%) and 2 comparison arms (7%).

**Fig. 1:**
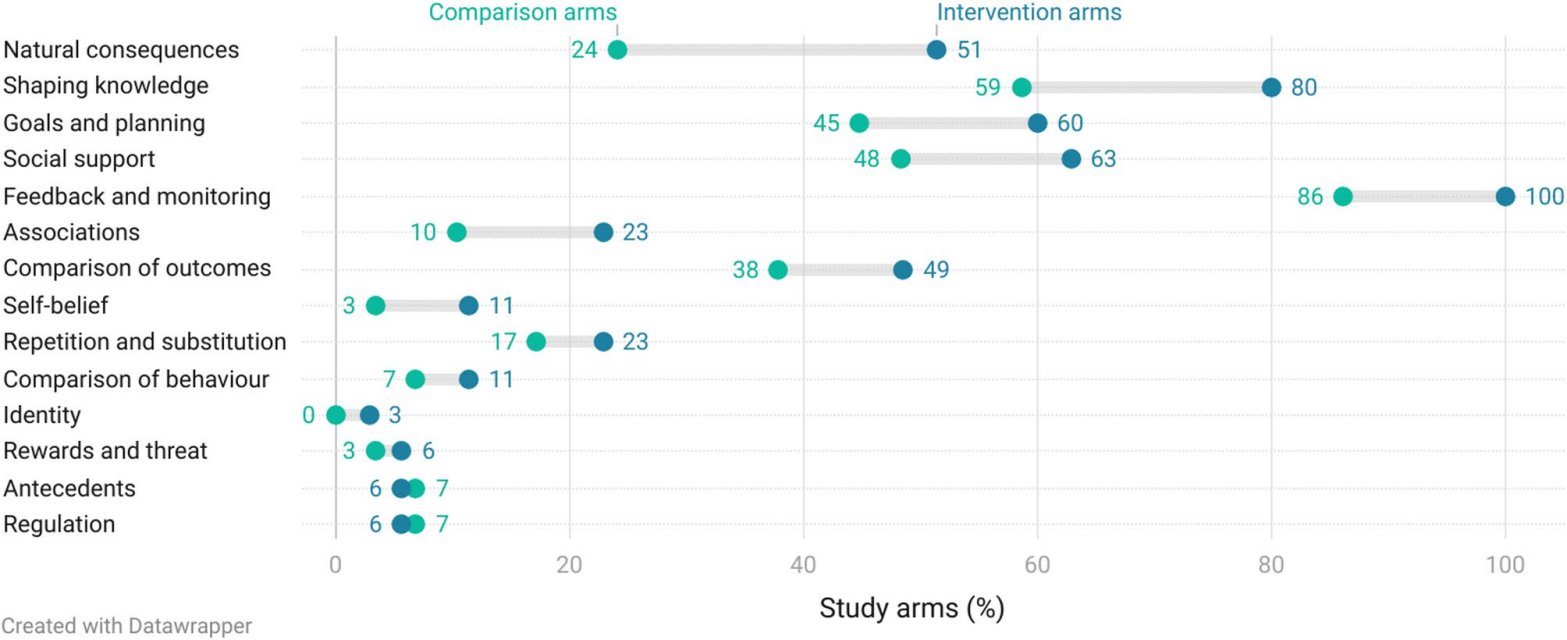
Category-level behaviour change techniques (BCTs) across intervention and comparison arms Each dot represents the number of unique arms applying at least one BCT from the corresponding group. The grey lines highlight the difference between comparison and intervention arms. Category labels in bold indicate statistically significant differences between groups (*p* < 0.05)

The frequency of BCT categories represented across intervention and comparison arms is outlined in Fig. 1. The most frequently used BCT categories within CGM-based biofeedback interventions were ‘*Feedback and monitoring*’ (*n* = 35/35, 100%), ‘*Shaping knowledg*e’ (*n* = 28/35, 80%), and ‘*Social support*’ (*n* = 22/35, 63%). After adjusting for multiple comparisons, the most pronounced difference between intervention and comparison arms was observed in the BCT category *‘Feedback and monitoring’*, with the BCT used in 100% of intervention arms compared to 86% in comparison arms (p-adjusted = 0.001). Similarly, *‘Shaping knowledge’* was used in 80% of intervention arms versus 59% in comparison arms (p-adjusted = 0.010), as was *‘Natural consequences’* (51% vs. 24%, p-adjusted = 0.001). Four categories (‘*Goals and planning*’, *‘Social support’*, ‘*Associations*’, and ‘*Self-belief*’) showed notable differences but did not reach statistical significance after adjustment for multiple comparisons (all p-adjusted ≥ 0.105). The remaining seven BCT categories showed no significant differences between arms (all p-adjusted ≥ 0.574).

**Fig. 2:**
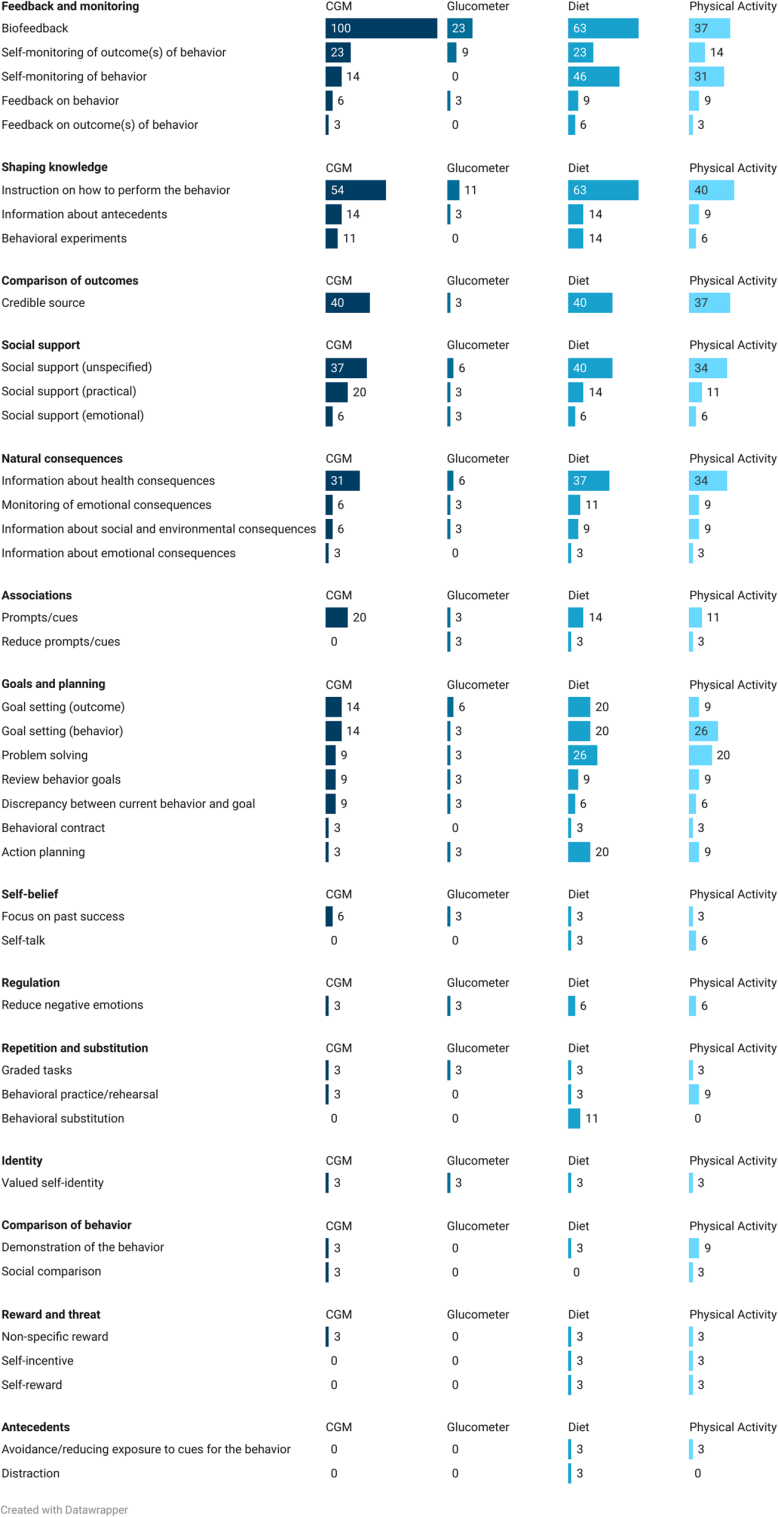
The percentage of BCT occurrences in the intervention arms (*n* = 35), categorized by behaviour change technique (BCT) category and target behaviours (CGM, glucometer, diet, and physical activity)

**Fig. 3:**
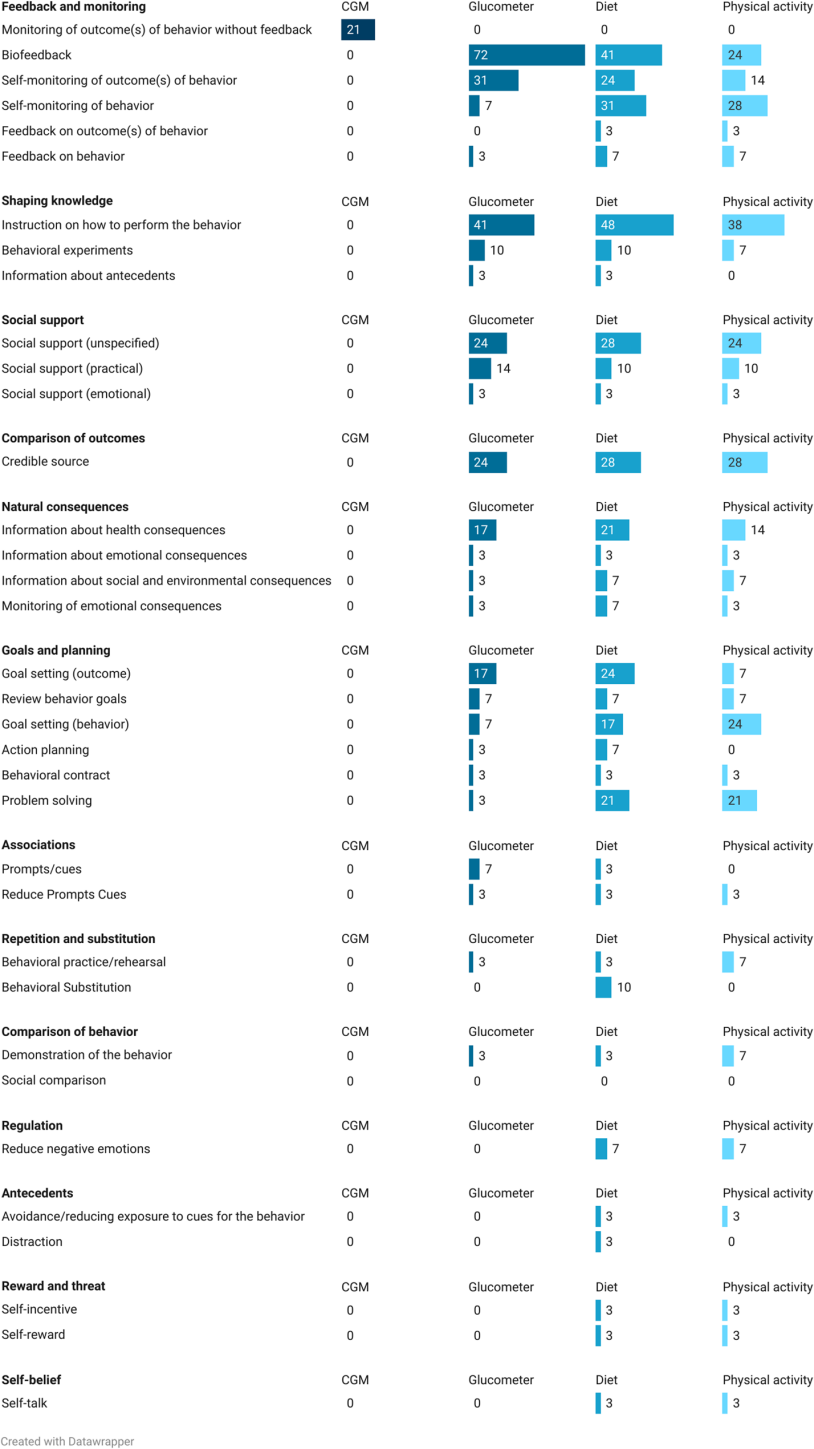
The percentage of BCT occurrences in the comparison arms (*n* = 29), categorized by behaviour change technique (BCT) category and target behaviours (CGM, glucometer, diet, and physical activity)

Within the categories, ‘*Feedback and monitoring*’, ‘*Shaping knowledg*e’, and ‘*Comparison of outcomes*’, the BCTs ‘*Biofeedback*’ (*n* = 35/35; 100%), ‘*Instruction on how to perform the behaviour’ (advise or agree on how to perform the behaviour)* (*n* = 19/35; 54%), and ‘*Credible source*’*(present verbal or visual communication from a credible source in favour of or against the behaviour)* (n = 14/35; 40%), respectively, were commonly reported in the intervention arms in relation to CGM use (Fig. 2). These three BCTs were also frequently used to prompt diet and PA changes, along with ‘*Self-monitoring of behaviour*’ *(establish a method for the person to monitor and record their behaviour(s) as part of a behaviour change strategy)* (diet: n = 16/35; 46%; PA: n = 11/35; 31%), ‘*Social support (unspecified)*’ *(advise on*,* arrange or provide social or noncontingent praise or reward for performance of the behaviour)* (diet: n = 14/35; 40%; PA: n = 12/35; 34%), and ‘*Information about health consequences*’ *(provide information (e.g. written*,* verbal*,* visual) about health consequences of performing the behaviour)* (diet: n = 13/35; 37%; PA: n = 12/35; 34%). When compared to the BCTs used within comparison arms (Fig. 3), ‘*Biofeedback*’ was frequently employed, but with the target of glucometer use rather than CGM use (n = 21/29; 72%). The only BCT that targeted CGM use in the comparison group was ‘*Monitoring of outcome(s) of behaviour without feedback*’ *(observe or record outcomes of behaviour with the person’s knowledge as part of a behaviour change strategy)* (n = 6/29; 21%), as participants using blinded CGM did not receive their glucose data. There were 6 BCTs that appeared in at least one intervention arm but were absent in all comparison arms. These included ‘*Discrepancy between current behaviour and goal*’ (n = 3/35; 9%), ‘*Focus on past success*’ (n = 2/35; 6%), ‘*Non-specific reward*’ (n = 1/35; 3%), ‘*Valued self-identity*’ (n = 1/35; 3%), ‘*Social comparison*’ (n = 1/35; 3%), and ‘*Graded tasks*’ (n = 1/35; 3%). Definitions and examples of use of each BCTs are available in Appendix 2. Coding Framework.

### Associations between behaviour change techniques and glycaemic control

The efficacy of CGM-based biofeedback interventions on HbA1c has been previously established [13]. To explore potential moderators of HbA1c changes, univariate linear regressions were conducted on 31 intervention arms with available HbA1c data. No individual BCTs or behavioural targets were significantly associated with HbA1c change (all *p* >0.05). Results, including effect estimates and confidence intervals, are presented in Table 1.

**Table 1:** Univariate linear regression results examining associations between individual BCTs or behavioural targets and change in HbA1c (*N* = 31 intervention arms)

	β	95% CI	*p*-value	*R*²
**BCT**				
2.3 Self-monitoring of behaviour	0.110	[−0.143, 0.364]	0.391	0.005
2.4 Self-monitoring of outcome(s) of behaviour	0.117	[−0.178, 0.412]	0.433	0.005
3.2 Social support (practical)	−0.088	[−0.438, 0.261]	0.617	0.002
4.1 Instruction on how to perform the behaviour	0.004	[−0.199, 0.206]	0.972	0.000
4.4 Behavioural experiments	0.106	[−0.304, 0.516]	0.611	0.002
1.1 Goal setting (behaviour)	−0.047	[−0.342, 0.249]	0.756	0.001
1.3 Goal setting (outcome)	0.020	[−0.308, 0.349]	0.902	0.000
3.1 Social support (unspecified)	−0.044	[−0.298, 0.210]	0.731	0.001
9.1 Credible source	0.017	[−0.217, 0.250]	0.887	0.000
5.1 Information about health consequences	−0.019	[−0.265, 0.227]	0.879	0.000
7.1 Prompts/cues	−0.209	[−0.535, 0.117]	0.207	0.012
4.2 Information about antecedents	−0.011	[−0.421, 0.400]	0.959	0.000
**Behavioural Target**				
Physical activity	−0.406	[−0.822, 0.01]	0.055	0.139
Glucometer use	−0.112	[−0.615, 0.392]	0.651	0.008
Medication adherence	−0.404	[−0.931, 0.123]	0.127	0.091

Dietary intake was excluded from the analysis as it was present across all studies, providing no variability for comparison

## Discussion

This study aimed to examine how CGM-based biofeedback is used in combination with other BCTs as part of a behaviour change intervention to help inform the development and reporting of future interventions in this space. Using the BCTTv1 taxonomy [9], and a classification of MoAs [21], we systematically identified and specified the target behaviours, behaviour change content, and MoAs within RCTs of CGM-based interventions.

Despite the documented efficacy of CGM-based interventions in improving glycaemic control, there was considerable heterogeneity in the use of BCTs across interventions. Intervention arms consistently included BCTs from the *‘Feedback and monitoring’* category, underscoring the central role of biofeedback. Other commonly used BCTs included *‘Instruction on how to perform the behaviour’ (Shaping knowledge’* category*)*, the use of a *‘Credible source’* (*‘Comparison of outcomes’* category), and *‘Social support’* (*‘Social support’* category), suggesting a focus on the role of providing education and external supports from trusted sources to facilitate CGM-based biofeedback as part of a comprehensive behaviour change strategy.

Notably, we observed a substantial overlap in the types of BCTs implemented across both intervention and comparison arms, although they differed in frequency and context. This suggests that certain BCTs - such as ‘*Biofeedback’*,* ‘Instruction on how to perform the behaviour’*, and *‘Credible source’* - are not unique to CGM-based strategies but may represent core components of broader behavioural interventions targeting glycaemic control. Furthermore, many of the most frequently used BCTs were employed across multiple behavioural targets, including CGM use, dietary change, and physical activity. This cross-cutting application implies that these BCTs may operate as generalizable mechanisms that support engagement across different health behaviours simultaneously. Moving forward, we may wish to consider these core BCTs listed above as a foundational cluster, particularly in combination with CGM-based biofeedback, given their relevance to enhancing motivation, self-regulation, and capability. Moreover, linking these core BCTs to specific MoAs could improve theoretical precision and promote consistency in intervention reporting and evaluation. Similar to our findings, a systematic review and meta-analysis of digital interventions to improve glycaemic control among those with type 2 diabetes, identified that ‘*Instruction on how to perform the behaviour’* was commonly used in intervention arms, and combinations of BCTs used across interventions arms were not able to be identified [62]. Notably, this meta-analysis did find that ‘*Self-monitoring of outcomes of behaviour*’ was statistically significantly associated with reduced HbA1c values. However, none of the included interventions in the aforementioned review used CGMs, so this finding may be more specific to interventions which use glucometers. In the present review, ‘*Self-monitoring of outcomes of behaviour’* is coded to describe intervention activities whereby participants were asked to manually enter and/or keep a log of their glucose values provided by their assigned device (CGM or glucometer) (see Appendix 2). Given the nature of CGMs, whereby glucose data is automatically stored, it is likely that this BCT functions differently and/or less effectively when applied to CGMs. Thus, we would not necessarily expect to replicate the previous finding in the present analysis, given the emphasis on CGMs. Indeed, this type of activity may be less salient at producing behaviour change in the context of CGMs where glycaemic values are automatically recorded, and there is much more information available either directly to the person or via a provider than when using a glucometer.

Our findings highlight the inconsistent application of behaviour change theory in CGM-based interventions, with few studies explicitly referencing theoretical frameworks or specifying target MoAs. This limits our ability to understand how and why specific BCTs contribute to behaviour change, and ultimately glycaemic improvements. This finding is not new in the context of reporting the theoretical bases of behaviour change interventions [27]. While there have been more than 700 reports of biological feedback in the published literature,^8^ there has been little study of the use of biological feedback as part of multi-component interventions with other BCTs. For example, a previous review of 277 multi-component behaviour change intervention reports found only six instances of the BCT ‘*Biofeedback’ *[21]. Further, few theories specify the role of biologically-based feedback as part of a behaviour change strategy, despite several theories reporting the role of feedback processes in behaviour change [63]. Existing research to classify the links between BCTs and MoAs identified that we have a limited understanding of how biofeedback works as a strategy to change behavior [10], with much of our existing knowledge being predicated on expert opinions and not empirical evidence of behaviour change [64]. Without more precise theoretical specification, we limit our ability to understand the ways in which biological feedback is similar to, or different from other forms of feedback on outcomes of behaviour. Future interventions would benefit from more systematic approaches to applying behaviour change theory to guide BCT selection, and enhanced reporting of intervention mechanisms. For example, specifying how CGM-based feedback interacts with cognitive and self-regulatory processes to help determine the extent to which biofeedback primarily supports self-efficacy, feedback and monitoring processes, habit formation or motivation for sustained behaviour change.

Again, although CGM-based biofeedback interventions are effective for glycaemic control, our univariate linear regression analysis did not identify any specific BCTs or targeted behaviours that were significantly associated with post HbA1c values. Few meta-analyses, and/or meta-regressions which examine the effects of specific BCTs, or combinations of BCTs identify significant pooled effects on measured outcomes, even those with larger numbers of included interventions [65, 66]. In studies that do identify significant pooled effects of BCTs on outcomes, the total number of BCTs identified in the systematic reviews have been smaller (e.g., 27–33 total BCTs), and evaluate larger numbers of included interventions (e.g., *n* = 45–143) [17, 26, 67]. Therefore the nature of our findings may be due to the high variability in intervention designs, the multifactorial nature of glycaemic control which may necessitate a greater range of BCTs needed for successful change, the potential for interactions between BCTs that were not captured in our analysis, or the possibility that the overall intervention context - rather than individual BCTs - drives effectiveness. Again, future research should explore whether particular combinations, sequences, or operationalizations of BCTs enhance intervention efficacy and whether individual differences (e.g., baseline self-regulation, diabetes self-management skills) influence responsiveness to specific BCTs. Further, whilst it is unlikely that 93 BCTs from BCTTv1 could be feasibly and operationally used in CGM interventions, based on the frequency of BCTs implemented within intervention arms, the findings do highlight the opportunity to consider a broader array of BCTs. A combination of BCTs may maximize the effectiveness of BCTs in supporting CGM-based interventions to reduce HbA1c. The heterogeneity of BCTs used in intervention arms coupled with the relatively small number of studies overall did not permit identification of such combinations of BCTs, similar to other meta-analyses evaluating complex, multi-component behaviour change interventions [68]. By including theory, and subsequently MoAs, in the development and evaluation of CGM-based interventions, there can be enhanced specification of how and why CGM-based interventions are effective, and further how and why certain combinations of techniques are effective, for which populations, and in which contexts.

The present study had several strengths. The most prominent is that it is the first study to systematically identify and characterise the behaviour change techniques used in CGM-based interventions, which we believe will be an important contribution to support the development and reporting of interventions leveraging CGM to change behaviour. There were several limitations present in the analysis potentially limiting the impact of the findings. The substantial heterogeneity in BCTs used within intervention arms and across studies, as well as our sample size limited our power to detect significant effcts and our ability to examine the effects of combinations of BCTs used in intervention arms. We had planned to conduct multiple linear regression using only the BCTs or behavioural targets that were significantly associated with HbA1c change in univariate models; however, as none met the significance threshold, we did not proceed with multivariable analysis. Similarly, we explored the use of factor analysis to identify clusters of BCTs but found that the limited number of studies and variation in BCT presence resulted in insufficient power for the models to converge meaningfully. A potential next step to this line of research would be to examine more specific implementation characteristics of CGM-based biofeedback studies, particularly those that describe the intensity and manner by which study participants are exposed to CGM-related BCTs (e.g., the frequency of biofeedback, the sequence in which BCTs are delivered, and the manner in which different behaviours are targeted). Furthermore, the variation in the content of the control arms limited our ability to assess the specific effects of BCTs used in combination with CGM-based biofeedback, versus glucometer-based biofeedback. We likely present an under-reporting of BCTs, as the process of extracting this data relied upon the extent to which study authors described the intervention content in specific detail. Most of the included studies were not published in behaviour change focused journals, where the specification of intervention content is more likely to be prioritized. Therefore, there is an enhanced likelihood that there were more BCTs present within intervention and or comparator arms, but which were not reported or described in sufficient detail to be extracted as specific BCTs. Where available we did consult supplementary files, protocol papers, clinical trial registries, and contact with the study authors to obtain more information about the interventions, however this information was not provided for all interventions. Future reports of research on CGM-based interventions should include more detailed, and/or intervention descriptions which are already specified by BCT to better facilitate the accumulation of knowledge about how CGM-based interventions are effective.

### Future directions

To enhance the effectiveness of CGM-based interventions, future research should prioritize evaluating specific BCT combinations and their MoAs. Our findings suggest that a core subset of BCTs - *‘Biofeedback’*,* ‘Instruction on how to perform the behaviour’*, and *‘Credible source’* - may serve as foundational elements across multiple behavioural targets within CGM interventions. Future evaluations should explore whether these techniques function synergistically when clustered together and whether they consistently operate through common MoAs such as enhancing self-efficacy, behavioural regulation, or motivation. Incorporating behaviour change theory into intervention development and specifying MoAs in the selection of BCTs will enhance the precision of future evaluations. Additionally, adhering to standardized reporting of intervention components, such as by using frameworks like the Template for Intervention Description and Replication (TIDieR) checklist [69], the BCTTv1 [9], and the Behaviour Change Intervention Ontology [19], could facilitate the accumulation of knowledge about how CGM-based interventions produce their effects.

## Conclusions

We have identified the number and type of BCTs used within randomized trials of interventions using CGM. We consider this an important step to call for better reporting of the active ingredients of these types of interventions and facilitate subsequent optimization, reproduction, scaling, and spread of effective versions of interventions using CGM. By systematically identifying the BCTs used in CGM-based interventions, this study highlights the need for greater transparency in intervention reporting, and for more theory-driven design and evaluation. Future work should prioritize evaluating specific BCT combinations and sequences along with their MoAs and evaluate their impact on glycaemic control and behaviours associated with blood glucose regulation.

## Supplementary Information

Supplementary Material 1.

Supplementary Material 2.

Supplementary Material 3.

## Data Availability

The datasets used and/or analysed during the current study are available from the corresponding author on reasonable request.

## Abbreviations

BCT: Behaviour change technique
BCTTv1: Behaviour Change Technique Taxonomy version 1
CGM: Continuous glucose monitor
HbA1c: Glycated haemoglobin
MoA: Mechanism of action
PA: Physical activity
RCT: Randomised controlled trial
SD: Standard deviation
