# Supplementary material for: Identifying behaviour change techniques within precision health interventions that use continuous glucose monitoring: a secondary analysis of a scoping review
Source: Int J Behav Nutr Phys Act. 2025 Nov 6;22:139. doi: 10.1186/s12966-025-01833-5 (PMC12590819; doi:10.1186/s12966-025-01833-5)
Supplement: Supplementary file 1 — Supplementary Material 1. [file 12966_2025_1833_MOESM1_ESM.docx]

**Appendix 2. Coding Framework**

| **Behaviour change technique (BCT)** | **BCT description** | **Examples from Intervention arms** | **Examples from Comparison arms** |
| --- | --- | --- | --- |
| **1.1 Goal setting (behaviour)** | Set or agree on a goal defined in terms of the behaviour to be achieved. | *The self-management support system consists of a webpage and mobile app for individuals to use during their daily life to set goals* [target behaviours: CGM, SMBG, PA, diet] (Ruissen et al., 2023) | *Participants in both groups were encouraged to solve problems and set diabetes-related goals* [target behaviours: PA, diet] (Allen et al., 2008) |
| **1.2 Problem solving** | Analyse, or prompt the person to analyse, factors influencing the behaviour and generate or select strategies that include overcoming barriers and/or increasing facilitators (includes ‘Relapse Prevention’ and ‘Coping Planning’). | *They were advised to note down the likely causes for patterns that were out of range and to suggest possible solutions in terms of changes to diet, activity, and insulin dose* [target behaviours: CGM, PA, diet, medication] (Murphy et al., 2008) | *[Education] focused on: coping with triggers and taking charge of one’s thoughts* [target behaviours: PA, diet] (Cox et al., 2020) |
| **1.3 Goal setting (outcome)** | Set or agree on a goal defined in terms of a positive outcome of wanted behaviour. | *American Diabetes Association programs use empowerment such*  *as goal setting techniques and contracting to enhance self-efficacy* [target behaviours: CGM, PA, diet, medication] (Meisenhelder-Smith, 2006) | *They were provided with specific targets: 3.5-5.3 mmol/L for fasting state; ≤ 7.8 mmol/L 1 hour after meals; ≤ 6.7 mmol/L 2 hours after meals* [target behaviour: SMBG] (Voormolen et al., 2018) |
| **1.4 Action planning** | Prompt detailed planning of performance of the behaviour (must include at least one of context, frequency, duration and intensity). Context may be environmental (physical or social) or internal (physical, emotional or cognitive) (includes ‘Implementation Intentions’). | *Participants were encouraged*  *to eat only when glucose levels were at or below a personalized*  *threshold. Personalized glucose thresholds were established*  *for each participant as the average of two consecutive morning glucose levels after fasting overnight*  *(8 hours or more)* [target behaviours: PA, diet] (Schembre et al., 2022) | *If glucose levels were higher than their cut-off, they were instructed to choose an activity that distracted them from food, and to wait at least 20 min before testing glucose again if still hungry.* [target behaviours: SMBG, diet] (Jospe et al., 2020) |
| **1.5 Review behaviour goal(s)** | Review behaviour goal(s) jointly with the person and consider modifying goal(s) or behaviour change strategy in light of achievement. This may lead to re-setting the same goal, a small change in that goal or setting a new goal instead of (or in addition to) the first, or no change. | *Have you made your goal sufficiently clear and realistic so that you can reach it with reasonable effort? If not, try to reformulate your goal and your activity planning and put them into practice.* [target behaviours: CGM, SMBG, PA, diet, medication] (Ruissen et al., 2023) | *Behavioural goals status, and educational needs were reviewed with patients by certified diabetes educators* [target behaviours: CGM, PA, diet, medication] (Meisenhelder-Smith, 2006) |
| **1.6 Discrepancy between current behaviour and goal** | Draw attention to discrepancies between a person’s current behaviour (in terms of the form, frequency, duration, or intensity of that behaviour) and the person’s previously set outcome goals, behavioural goals or action plans (goes beyond self-monitoring of behaviour). | *It seems as if it is difficult to reach your goal. Can it be that you have set your goal too high? Often*  *one is more successful with smaller intermediate steps. Consider whether it is useful to reduce*  *your goal in such a way that you need to achieve the goal a bit longer, the goal is challenging for*  *you, but still achievable in everyday life.* [target behaviours: CGM, SMBG, PA, diet, medication] (Ruissen et al., 2023) | N/A |
| **1.8 Behavioural contract** | Create a written specification of the behaviour to be performed, agreed on by the person, and witnessed by another. | *Demonstrate commitment to change through written contract.* [target behaviours: CGM, PA, diet, medication] (Meisenhelder-Smith, 2006) | *Demonstrate commitment to change through written contract.* [target behaviours: SMBG, PA, diet, medication] (Meisenhelder-Smith, 2006) |
| **2.2 Feedback on behaviour** | Monitor and provide informative or evaluative feedback on performance of the behaviour (e.g. form, frequency, duration, intensity). | *Continual monitoring*  *of the integrated health care data (blood glucose, CGM metrics, body weight, blood pressure, exercise, and diet) by medical staff and personal educational feedback via text messages were provided only to those in group C.* [target behaviours: CGM, PA, diet] (You-Bin et al., 2023) | *The dietitian provided feedback on the logs [of food intake and physical activity] to help participants achieve their goals.* [target behaviours: PA, diet] (Cox et al., 2020) |
| **2.3 Self-monitoring of behaviour** | Establish a method for the person to monitor and record their behaviour(s) as part of a behaviour change strategy. | *The patients were also*  *asked to record glucose values, time and contents of meals, insulin injections, exercise periods and symptomatic hypoglycaemic events in a logbook.* [target behaviours: CGM, PA, diet] (Alfadhli et al., 2016) | *Patients were also asked to keep a diary that recorded the amount and type of food consumed every day.* [target behaviours: diet] (Sato et al., 2016) |
| **2.4 Self-monitoring of outcome(s) of behaviour** | Establish a method for the person to monitor and record the outcome(s) of their behaviour as part of a behaviour change strategy. | *Breakfast challenge learning module – Track your glucose when you wake up and your peak glucose* [target behaviours: CGM] (Price et al., 2021) | *All participants were instructed to self-monitor their blood glucose at least four times daily* [target behaviours: SMBG] (Aronson et al., 2022) |
| **2.5 Monitoring of outcome(s) of behaviour without feedback** | Observe or record outcomes of behaviour with the person’s knowledge as part of a behaviour change strategy. | N/A | *For the blinded flash glucose (FGM) monitoring group, the results of blood glucose in blinded FGM were masked, and patients could take SMBG at any time.* [target behaviours: CGM] (Yan et al., 2022) |
| **2.6 Biofeedback** | Provide feedback about the body (e.g. physiological or biochemical state) using an external monitoring device as part of a behaviour change strategy. | *The scanning group used the Freestyle Libre Flash Glucose Monitoring system (Abbott Diabetes Care, Australia), which continuously measures interstitial glucose every 15 min.* [target behaviours: CGM] (Jospe et al., 2020) | [*Participants] were given glucometers (FreeStyle Optimum Neo; Abbott Diabetes Care), lancets, glucose test strips sufficient for BGM twice daily* [target behaviours: SMBG] (Choe et al., 2022) |
| **2.7 Feedback on outcome(s) of behaviour** | Monitor and provide feedback on the outcome of performance of the behaviour. | *Continual monitoring of the integrated health care data (blood glucose, CGM metrics, body weight, blood pressure, exercise, and diet) by medical staff and personal educational feedback via text messages were provided only to those in group C.* [target behaviours: CGM, PA, diet] (You-Bin et al., 2023) | *The SMBG group was also instructed about their diet and exercise habits every month based on the SMBG values for 1 month* [target behaviours: PA, diet] (Yoo et al., 2008) |
| **3.1 Social support (unspecified)** | Advise on, arrange or provide social  support (e.g. from friends, relatives,  colleagues,’ buddies’ or staff) or noncontingent praise or reward for  performance of the behaviour. It includes encouragement and counselling, but only when it is directed at the behaviour. | *At these visits, participants’ glucose control was reviewed with an HCP and the effects of diet/lifestyle on glucose trends and insulin dose modifications were discussed.* [target behaviours: CGM, diet] (Haak et al., 2017) | *Control group participants continued to receive usual clinical care and had usual care visits every 3 months. In Australian primary care, usual clinical care for people with type 2 diabetes varies between practices. Some GPs adopt a more systematic, team-based and organised approach, whereas others embed care within routine consultations.* [target behaviours: SMBG, PA, diet] (Furler et al., 2020) |
| **3.2 Social support (practical)** | Advise on, arrange, or provide practical help (e.g. from friends, relatives, colleagues, ‘buddies’ or staff) for performance of the behaviour. | *Contact details for the research nurse were provided to participants for remote device support and to answer any queries on insertion, initiation and changing of the sensor* [target behaviours: CGM] (Taylor et al., 2019) | *There were phone visits with a study-site clinician at weeks 2, 6 and 10 in both groups to review the SMBG data… During these calls, there were structured discussions about what the subjects learned from their glucose monitoring, what changes were made in response to the data, and what the study clinician observed.* [target behaviours: SMBG] (Price et al., 2021) |
| **3.3 Social support (emotional)** | Advise on, arrange, or provide emotional social support (e.g. from friends, relatives, colleagues, ‘buddies’ or staff) for  performance of the behaviour. | *It seems as if it was difficult to achieve your goal in the (#review-period). This might have been just an ''off-day'' or ''off-week''. But maybe you feel that something is hindering you in monitoring your*  *(#ODL). It can help to discuss this with others. Do you know anybody with whom you can share*  *questions or difficulties with regard to monitoring your (#ODL)? This can be a family member, friend, caregiver or maybe even a forum on the internet.* [target behaviours: CGM, SMBG, PA, diet, medication] (Ruissen et al., 2023) | *[Focus is] placed on developing skills and self-confidence. To foster self-confidence the diabetes educators instructs the patient on the correct self-care skills then allows the participants to return the demonstration.* [target behaviours: SMBG, PA, diet, medication] (Meisenhelder-Smith, 2006) |
| **4.1 Instruction on how to perform the behaviour** | Advise or agree on how to perform the behaviour (includes ‘Skills training’). | *In the 2nd week, the flash glucose monitoring (FGM) group received directions on the best use of FGM, including meanings of the trend arrows and glucose profiles for treatment adjustment, and how to deal with hyperglycaemia and hypoglycaemia.* [target behaviours: CGM] (Zhang et al., 2021) | *The materials comprised four themes, including basic knowledge of T2DM, reasonable diets for T2DM, exercise therapy for T2DM and the prevention of T2DM complications.* [target behaviours: PA, diet] (Guo et al., 2023) |
| **4.2 Information about antecedents** | Provide information about antecedents (e.g. social and environmental situations and events, emotions, cognitions) that reliably predict performance of the behaviour. | *They were advised to note down the likely causes for patterns that were out of range and to suggest possible solutions in terms of changes to diet, activity, and insulin dose* [target behaviours: CGM, PA, diet] (Murphy et al., 2008) | *During months 2–6, participants did not measure their glucose and instead were encouraged to rely on their sensations of hunger [hunger training] to guide their eating, which they had developed in month 1.* [target behaviours: SMBG, diet] (Jospe et al., 2020) |
| **4.4 Behavioural experiments** | Advise on how to identify and test  hypotheses about the behaviour, its causes and consequences, by collecting and interpreting data. | *Breakfast challenge learning module – For the next week, eat as many different breakfasts as you normally do… track your glucose when you wake up and your peak glucose* [target behaviours: CGM, diet] (Price et al., 2021) | *In the week following session*  *1, participants monitored their routine blood glucose (BG)-relevant choices in their diary… they noted the impact these choices had on their BG.* [target behaviours: SMBG, PA, diet] (Cox et al., 2020) |
| **5.1 Information about health consequences** | Provide information (e.g. written, verbal, visual) about health consequences of performing the behaviour. | *Provided dietary education, carbohydrate counting, basic rules to prevent and correct hypo- and hyperglycaemia… All the participants had their knowledge assessed monthly with the support of a dietician and a nurse.* [target behaviours: CGM, PA, diet] (Tumminia et al., 2021) | *The materials comprised four themes, including basic knowledge of T2DM, reasonable diets for T2DM, exercise therapy for T2DM and the prevention of T2DM complications.* [target behaviours: PA, diet] (Guo et al., 2023) |
| **5.3 Information about social and environmental consequences** | Provide information (e.g. written, verbal, visual) about social and environmental consequences of performing the behaviour. | *For those who have low scores on perceived benefits of care, the educator can emphasize the immediate and long-term rewards for controlling diabetes. Avoiding pain, financial expense, and other problems due to poor management are stressed.* [target behaviours: CGM, PA, diet, medication] (Meisenhelder-Smith, 2006) | *For those who have low scores on perceived benefits of care, the educator can emphasize the immediate and long-term rewards for controlling diabetes. Avoiding pain, financial expense, and other problems due to poor management are stressed.* [target behaviours: CGM, PA, diet, medication] (Meisenhelder-Smith, 2006) |
| **5.4 Monitoring of emotional consequences** | Prompt assessment of feelings after  attempts at performing the behaviour. | *As part of the CGM Diary - self-evaluation for the day (Scores: 1 [Red frowning face] – 5) [Green smiley face])* [target behaviours: SMBG] (Choe et al., 2022) | *[Linking the] association of their feelings of hunger with glucose levels* [target behaviours: diet] (Jospe et al., 2020) |
| **5.6 Information about emotional consequences** | Provide information (e.g. written, verbal, visual) about emotional consequences of performing the behaviour | *For those who have low scores on perceived benefits of care, the educator can emphasize the immediate and long-term rewards for controlling diabetes… Benefits of increased sense of well-being, feeling good, and having the energy to enjoy life are emphasized* [target behaviours: CGM, PA, diet, medication] (Meisenhelder-Smith, 2006) | *For those who have low scores on perceived benefits of care, the educator can emphasize the immediate and long-term rewards for controlling diabetes… Benefits of increased sense of well-being, feeling good, and having the energy to enjoy life are emphasized* [target behaviours: CGM, PA, diet, medication] (Meisenhelder-Smith, 2006) |
| **6.1 Demonstration of the behaviour** | Provide an observable sample of the performance of the behaviour, directly in person or indirectly e.g. via film, pictures, for the person to aspire to or imitate (includes ‘Modelling’). | *Role playing was done to reinforce [physical activity] skills* [target behaviours: PA] (Allen et al., 2011) | *Skills are taught using a variety of techniques including verbal instruction, demonstration, problem solving, and empowerment training.* [target behaviours: SMBG, PA, diet, medication] (Meisenhelder-Smith, 2006) |
| **6.2 Social comparison** | Draw attention to others’ performance to allow comparison with the person’s own  performance | *The first step provided*  *not only feedback on the participant’s own expected areas of activity-related glucose reduction (performance accomplishment), but also examples of CGMS graphs and stories from role models to show activity-related glucose reductions (vicarious experience).* [target behaviours: CGM, PA] (Allen et al., 2008) | N/A |
| **7.1 Prompts/cues** | Introduce or define environmental or social stimulus with the purpose of prompting or cueing the behaviour. The prompt or cue would normally occur at  the time or place of performance | *The processor could analyse the types and amounts of food that the patient had consumed within two hours before the fluctuation and combine this with previous data to propose which food or which foods could cause such changes. The processor could then remind GPs and the patient that the food could seriously affect the patient’s blood glucose.* [target behaviours: CGM, PA, diet] (Guo et al., 2023) | *Participants were sent a text message every month to remind them to fill in their booklets.* [target behaviours: SMBG, diet] (Jospe et al., 2020) |
| **7.3 Reduce prompts cues** | Withdraw gradually prompts to perform the behaviour (includes ‘Fading’) | *They inserted one 7-day Dexcom G5 sensor at each treatment session, and another 8 weeks after the last treatment session. After the sensors inserted between classes 2–3 and 3–4 expired, participants were encouraged to rely on BG monitoring to note changes in their BG. This was done to promote independence from CGM.* [target behaviours: CGM] (Cox et al., 2020) | *They inserted one 7-day Dexcom G5 sensor at each treatment session, and another 8 weeks after the last treatment session. After the sensors inserted between classes 2–3 and 3–4 expired, participants were encouraged to rely on BG monitoring to note changes in their BG. This was done to promote independence from CGM.* [target behaviours: CGM] (Cox et al., 2020) |
| **8.1 Behavioural practice/rehearsal** | Prompt practice or rehearsal of the performance of the behaviour one or more times in a context or at a time when the performance may not be necessary, in order to increase habit and skill. | *The program not only focuses on increasing knowledge of self-care activities, but provides time for practicing these skills.* [target behaviours: CGM, PA, diet, medication] (Meisenhelder-Smith, 2006) | *The program not only focuses on increasing knowledge of self-care activities, but provides time for practicing these skills.* [target behaviours: SMBG, PA, diet, medication] (Meisenhelder-Smith, 2006) |
| **8.2 Behavioural substitution** | Prompt substitution of the unwanted behaviour with a wanted or neutral behaviour. | *A low (glucose index) GI and glucose nutrition education focused on teaching participants to swap high GI and glucose food to foods with lower GI and glucose* [target behaviours: diet] Cheekma et al., 2022) | *Participants were provided with education on food exchanges and provided lists of alternative foods to assist participants with making*  *suitable food substitutions to improve their dietary flexibility.* [target behaviours: diet] (Taylor et al., 2019) |
| **9.1 Credible source** | Present verbal or visual communication from a credible source in favour of or against the behaviour. | *At each study visit, SMBG data and FGM profiles were reviewed by a physician, who prescribed the appropriate therapeutic modifications.* [target behaviours: CGM, PA, diet] (Tumminia et al., 2021) | *For participants*  *in the usual care group, regular care visits with their usual*  *diabetes care team were continued* [target behaviours: SMBG] (Ruissen et al., 2023) |
| **10.3 Non-specific reward** | Arrange delivery of a reward if and only if there has been effort and/or progress in performing the behaviour (includes  ‘Positive reinforcement’) | *Encouragement [via text message]:*  *Weight loss (0.0) kg, good result;*  *Fasting glucose level, within the target range; Walk more than 10,000 steps, practice this week as well.* [target behaviours: PA, diet] (You-Bin et al., 2023) | N/A |
| **10.7 Self-incentive** | Plan to reward self in future if and only if  there has been effort and/or progress in performing the behaviour. | *Discuss personal incentives and*  *rewards.* [target behaviours: CGM, PA, diet, medication] (Meisenhelder-Smith, 2006) | *Discuss personal incentives and*  *rewards.* [target behaviours: SMBG, PA, diet, medication] (Meisenhelder-Smith, 2006) |
| **10.9 Self-reward** | Prompt self-praise or self-reward if and only if there has been effort and/or progress in performing the behaviour. | *Discuss personal incentives and*  *rewards.* [target behaviours: CGM, PA, diet, medication] (Meisenhelder-Smith, 2006) | *Discuss personal incentives and*  *rewards.* [target behaviours: SMBG, PA, diet, medication] (Meisenhelder-Smith, 2006) |
| **11.2 Reduce negative emotions** | Advise on ways of reducing negative  emotions to facilitate performance of the behaviour (includes ‘Stress Management’). | *Can you plan more breaks? Even a small 1-5 min. break can be very effective in reducing stress. Try to plan a small break every hour.* [target behaviours: CGM, SMBG, PA, diet, medication] (Ruissen et al., 2023) | *[Education] focused on: coping with triggers and taking charge of one’s thoughts* [target behaviours: PA, diet] (Cox et al., 2020) |
| **12.4 Distraction** | Advise or arrange to use an alternative focus for attention to avoid triggers for unwanted behaviour. | *If glucose levels were higher than their cut-off, they were instructed to choose an activity that distracted them from food, and to wait at least 20 min before testing*  *glucose again if still hungry.* [target behaviours: diet] (Jospe et al., 2020) | *If glucose levels were higher than their cut-off, they were instructed to choose an activity that distracted them from food, and to wait at least 20 min before testing*  *glucose again if still hungry.* [target behaviours: diet] (Jospe et al., 2020) |
| **13.4 Valued self-identity** | Advise the person to write or complete rating scales about a cherished value or personal strength as a means of affirming the person’s identity as part of a behaviour change strategy (includes ‘Self-affirmation’) | *Even small successes count! It might be good to think about what is important in your life. This is what you have said what is important for you* [target behaviours: CGM, SMBG, PA, diet, medication] (Ruissen et al., 2023) | N/A |
| **15.3 Focus on past success** | Advise to think about or list previous successes in performing the behaviour (or parts of it). | *Women were asked firstly to note the proportion of time spent with glucose values within the target range, making efforts to focus attention on the positive aspects of each continuous or intermittent capillary glucose meter profile* [target behaviours: CGM] (Murphy et al., 2008) | N/A |
| **15.4 Self-talk** | Prompt positive self-talk (aloud or silently) before and during the behaviour. | *Modules on problem-solving techniques [to support PA] were developed including… (7) self-talk* [target behaviours: PA] (Allen et al., 2011) | N/A |

**Notes.**

No BCTs were coded for two BCT categories: 14. Scheduled consequences and 16. Covert learning.

CGM = continuous glucose monitoring, PA = physical activity, SMBG = self-monitoring blood glucose
