# Supplementary material for: Identifying behaviour change techniques within precision health interventions that use continuous glucose monitoring: a secondary analysis of a scoping review
Source: Int J Behav Nutr Phys Act. 2025 Nov 6;22:139. doi: 10.1186/s12966-025-01833-5 (PMC12590819; doi:10.1186/s12966-025-01833-5)
Supplement: Supplementary file 2 — Supplementary Material 2. [file 12966_2025_1833_MOESM2_ESM.docx]

**Appendix 1. BCT Coding Rules**

1. Definition of a BCT**:** A replicable component of an intervention designed to alter or redirect causal processes that regulate behaviour; that is, a technique is proposed to be an ‘active ingredient’ (e.g. feedback, self-monitoring, and reinforcement).
   - 1. BCTs contain verbs (*e.g., provide, advise, arrange*) that refer to the action(s)
        taken by the person/s delivering the technique. BCTs can be delivered by an 'interventionist' or self-delivered.
     2. BCTs contain the term "behaviour" referring to a single action or sequence of actions that includes the performance of wanted behaviour(s) and/or inhibition (non-performance) of unwanted behaviour(s).
2. Coders should familiarise themselves with BCTs *(e.g., labels, definitions and examples), and should r*ead and re-read BCT definitions as many times as needed (i.e. to have a good understanding of what must be specified in the intervention description in order for the BCT to be coded). The whole intervention description should be read before beginning to code BCTs. There may be multiple BCTs within one sentence.
3. Where BCTs have not already been coded within papers, the most recent BCT taxonomy of 93 BCTs, BCTTv1 ([Michie et al., 2013](#_heading=h.30j0zll)), should be used. Where BCTs have been coded using an earlier version of the taxonomy, these will be re-coded using BCTTv1 to standardise coding. BCTs that are the same across taxonomies will not be recoded, provided coders agree with the previous coding. If the intervention was previously coded by a review, the paper should still be re-coded as we do not have data on *where* the BCTs were coded within the intervention papers.
4. BCTs should only be coded if they are targeting one or more of the target behaviours or key preparatory behaviours of the intervention - not supportive behaviours that are active ingredients in *engaging* the person. Thus, while we would include ‘condom buying’ in a safe sex intervention, we would not include ‘parent/child’ communication in a dietary intervention (where the behaviour measured is fruit & veg intake). This is due to (a) the complexity of this process, (b) the time constraints of the project and (c) the lack of clarity afforded to causal sequences within intervention descriptions.
   - 1. If unsure whether ‘preparatory’ behaviour or not, keep aside for further review
     2. Code all BCTs which are to promote use of CGM (target behaviour = CGM)
        1. Do not code additional rows to show that these BCTs are also used to change diet/PA
     3. Code all other BCTs which are to change diet and PA
     4. Do not code implementation related BCTs like this: “During the study period, the registered nurse will make phone/remote contacts every four weeks to encourage both the intervention group and the control group to continue the lifestyle modification.” – only specific to CGM/GM not to lifestyle changes
     5. Make sure to check the outcome measures section to see if they measure behaviour or if the behaviour should be e.g., lifestyle behaviours or unspecified behaviour
5. If the intervention description includes a label from the BCT taxonomy (e.g. ‘problem solving’), but the description of this BCT appears to contradict the definition from the BCT taxonomy (e.g. ‘problem solving activities that asked participants to decide if statements were true or false’), do not code as problem solving.
6. **Do not** code ‘Adding objects’ for providing a glucose monitor, **do** code ‘adding objects’ when providing an app (or some tool) to view the feedback (blinded vs unblinded CGM).
7. If CGM is blinded, and no feedback is provided, do not code ‘Biofeedback’, code as ‘Monitoring of outcome(s) without feedback.
8. Do not code BCTs which were applied pre-randomization.
9. Be on the lookout for papers which are using SBGM in conjunction with CGM, this is typically for older CGM models and this is for calibration purposes and not for behaviour change purposes.
10. Do not code pharmacological support when insulin modifications are part of the CGM strategy.

**Mechanisms of Action: Guidelines**

1. Definition of a Mechanism of Action: The process through which a BCT affects behaviour.
2. For each BCT, examine for presence of link to a mechanism of action (see Table 1 below). In order to be coded as a link, the author must hypothesise that BCT(s) X changes behaviour through Mechanism(s) Y, where Mechanism Y is specified as a mechanism of action (i.e. there must be an explicit link to behaviour).

*Table 1:* Coding BCT-mechanism of action links

| **CODE Code as Mechanism of Action if:** | **Do NOT code as Mechanism of Action if:** |
| --- | --- |
| - It is labelled a ‘determinant’ or ‘mediator’ of behaviour - It is explicitly hypothesised to change behaviour (wording includes: ‘influences’, ‘has an effect on’/’affects’, ‘changes’) | - Authors have only described the mechanism of action in relation to previous research (not in context of current study) - It is unclear whether a construct is an mechanism of action or a BCT - A construct is specified as a mechanism of action, but the measurement of the construct is a measure of the degree of implementation of, or engagement with, a BCT, rather than a mediator between the BCT and behaviour (see Figure 1 below). For example, action planning is said to have an influence on behaviour and is measured by asking participants whether or not they set an action plan. - A theory is stated as a theory of behaviour change, and the construct in question is termed a ‘key construct’ of that theory, but not specified to be a ‘key construct’ for behaviour change. |
| **Code as Link if:** | **Do not code as link if:** |
| - One BCT has been explicitly hypothesised to link to one or more mechanisms of action, or one mechanism of action has been explicitly hypothesised to link to one or more BCTs.   **Example:**   - ‘We hypothesised that attitude towards the behaviour would mediate the effect of the normative feedback (containing BCTs X, Y & Z) on behaviour’. | - Two or more BCTs are linked to 2 or more mechanisms of action, and there are no 1-1 or n-1 links.   - If authors clearly state 2 BCTs work through both of 2 MoAs, ok to include (e.g. authors state that BCT X and BCT Y both influence behaviour through both of MoA A and MoA B).   - Note 5a coding guideline.   **Example:**   - Table with groups of BCTs linked to groups of mechanisms of action. - ‘Social norms, attitude and self-efficacy should mediate effect of the the social comparison and social support components’ |
