# Supplementary material for: Identifying behaviour change techniques within precision health interventions that use continuous glucose monitoring: a secondary analysis of a scoping review
Source: Int J Behav Nutr Phys Act. 2025 Nov 6;22:139. doi: 10.1186/s12966-025-01833-5 (PMC12590819; doi:10.1186/s12966-025-01833-5)
Supplement: Supplementary file 3 — Supplementary Material 3. [file 12966_2025_1833_MOESM3_ESM.docx]

**Appendix 3. Study Characteristics**

| **Author, Year** | **Overview of study arms** | **Targeted behaviour(s)** | **BCTs** |
| --- | --- | --- | --- |
| **Intervention arms** | | | |
| Ahn, 2023 (Intervention #1) | - Unblinded CGM - Conventional care, including education on activity and medication adherence - Individual and group dietary coaching - Diet tracking | CGM | 2.6 Biofeedback  3.1 Social support (unspecified)  9.1 Credible source |
|  |  | Diet | 1.1 Goal setting (behaviour)  1.4 Action planning  2.3 Self-monitoring of behaviour  2.7 Feedback on outcome(s) of behaviour  3.1 Social support (unspecified)  4.1 Instruction on how to perform the behaviour  9.1 Credible source |
|  |  | Activity | 5.1 Information about health consequences |
| Ahn, 2023 (Intervention #2) | - Unblinded CGM - Conventional care, including education on activity and medication adherence | CGM | 2.6 Biofeedback |
|  |  | Activity | 5.1 Information about health consequences |
| Alfadhli, 2016 | - Unblinded CGM - Glucometer - Glucose tracking - Diet tracking - Activity tracking - Medication tracking - Treatment adjustments based on CGM data | CGM | 1.3 Goal setting (outcome)  2.3 Self-monitoring of behaviour  2.4 Self-monitoring of outcome(s) of behaviour  2.6 Biofeedback |
|  |  | Diet | 1.3 Goal setting (outcome)  2.3 Self-monitoring of behaviour  2.4 Self-monitoring of outcome(s) of behaviour |
| Allen, 2008 | - Unblinded CGM - Activity monitor - Diabetes education - CGM counselling relative to physical activity - Prescription for physical activity program | CGM | 2.6 Biofeedback  3.2 Social support (practical)  4.1 Instruction on how to perform the behaviour  6.2 Social comparison |
|  |  | Diet | 1.1 Goal setting (behaviour)  1.2 Problem solving  2.6 Biofeedback  3.2 Social support (practical)  4.1 Instruction on how to perform the behaviour |
|  |  | Activity | 1.1 Goal setting (behaviour)  1.2 Problem solving  1.4 Action planning  2.6 Biofeedback  3.2 Social support (practical)  4.1 Instruction on how to perform the behaviour  5.1 Information about health consequences  6.2 Social comparison |
| Allen, 2011 (Intervention #1) | - CGM (blinding unclear) - CGM counselling on activity - Prescription for physical activity program - Problem-solving counselling | CGM | 1.1 Goal setting (behaviour)  2.3 Self-monitoring of behaviour  2.6 Biofeedback  9.1 Credible source |
|  |  | Diet | 2.3 Self-monitoring of behaviour |
|  |  | Activity | 1.1 Goal setting (behaviour)  1.2 Problem solving  2.3 Self-monitoring of behaviour  2.6 Biofeedback  3.1 Social support (unspecified)  4.1 Instruction on how to perform the behaviour  6.1 Demonstration of the behaviour  8.1 Behavioural practice / rehearsal  9.1 Credible source  15.4 Self-talk |
| Allen, 2011 (Intervention #2) | - CGM (blinding unclear) - CGM counselling on activity - Prescription for physical activity program - Attention-control diabetes education | CGM | 1.1 Goal setting (behaviour)  2.3 Self-monitoring of behaviour  2.6 Biofeedback  4.1 Instruction on how to perform the behaviour  9.1 Credible source |
|  |  | Diet | 2.3 Self-monitoring of behaviour |
|  |  | Activity | 1.1 Goal setting (behaviour)  2.3 Self-monitoring of behaviour |
| Aronson, 2023 | - Unblinded CGM - Diabetes self-management education | CGM | 1.1 Goal setting (behaviour)  1.5 Review behaviour goals  2.3 Self-monitoring of behaviour  2.4 Self-monitoring of outcome(s) of behaviour  2.6 Biofeedback  3.1 Social support (unspecified)  4.1 Instruction on how to perform the behaviour  4.4 Behavioural experiments  5.1 Information about health consequences  5.4 Monitoring of emotional consequences  7.1 Prompts/cues  9.1 Credible source |
|  |  | Diet | 1.1 Goal setting (behaviour)  1.5 Review behaviour goals  2.3 Self-monitoring of behaviour  2.4 Self-monitoring of outcome(s) of behaviour  2.6 Biofeedback  3.1 Social support (unspecified)  4.1 Instruction on how to perform the behaviour  4.4 Behavioural experiments  5.1 Information about health consequences  5.4 Monitoring of emotional consequences  9.1 Credible source |
|  |  | Activity | 1.1 Goal setting (behaviour)  1.5 Review behaviour goals  2.3 Self-monitoring of behaviour  2.4 Self-monitoring of outcome(s) of behaviour  2.6 Biofeedback  3.1 Social support (unspecified)  4.1 Instruction on how to perform the behaviour  4.4 Behavioural experiments  5.1 Information about health consequences  5.4 Monitoring of emotional consequences  9.1 Credible source |
|  |  | Medication adherence | 1.1 Goal setting (behaviour)  1.5 Review behaviour goals  2.3 Self-monitoring of behaviour  2.4 Self-monitoring of outcome(s) of behaviour  2.6 Biofeedback  3.1 Social support (unspecified)  4.1 Instruction on how to perform the behaviour  4.4 Behavioural experiments  5.1 Information about health consequences  5.4 Monitoring of emotional consequences  9.1 Credible source |
| Chekima, 2022 | - Unblinded CGM - High-, moderate-, and low-GI meals followed by a review of CGM data - Nutrition education on low glycaemic index and glycaemic load foods | CGM | 2.6 Biofeedback |
|  |  | Diet | 2.6 Biofeedback  5.1 Information about health consequences  8.2 Behavioural substitution |
| Chekima, 2022 | - Unblinded CGM - Nutrition education on low glycaemic index and glycaemic load foods | CGM | 2.6 Biofeedback  4.1 Instruction on how to perform the behaviour |
|  |  | Diet | 1.2 Problem solving  1.4 Action planning  4.1 Instruction on how to perform the behaviour  8.2 Behavioural substitution |
| Choe, 2022 | - Unblinded CGM - Structured education using SEOUL algorithm to encourage healthy meals that result in low postprandial glucose levels | CGM | 2.3 Self-monitoring of behaviour  2.4 Self-monitoring of outcome(s) of behaviour  2.6 Biofeedback  3.2 Social support (practical)  4.1 Instruction on how to perform the behaviour |
|  |  | Diet | 1.2 Problem solving  2.3 Self-monitoring of behaviour  2.4 Self-monitoring of outcome(s) of behaviour  2.6 Biofeedback  3.2 Social support (practical)  4.1 Instruction on how to perform the behaviour  4.4 Behavioural experiments  5.4 Monitoring of emotional consequences |
|  |  | Activity | 2.3 Self-monitoring of behaviour |
| Cosson, 2009 | - Blinded CGM - Treatment adjustments based on CGM data | CGM | 2.6 Biofeedback  3.2 Social support (practical)  4.1 Instruction on how to perform the behaviour  5.1 Information about health consequences |
|  |  | Diet | 3.2 Social support (practical)  4.1 Instruction on how to perform the behaviour  5.1 Information about health consequences |
|  |  | Activity | 3.2 Social support (practical)  4.1 Instruction on how to perform the behaviour  5.1 Information about health consequences |
| Cox, 2020 | - Unblinded CGM - Glucometer - Education on low-glycaemic load foods, increasing physical activity, and decreasing sedentary time - Diet tracking - Activity tracking - Glucose tracking | CGM | 2.6 Biofeedback  3.1 Social support (unspecified)  4.1 Instruction on how to perform the behaviour  4.4 Behavioural experiments  5.1 Information about health consequences  7.1 Prompts / cues  9.1 Credible source |
|  |  | Glucometer | 2.6 Biofeedback  7.3 Reduce prompts / cues |
|  |  | Diet | 1.2 Problem solving  1.3 Goal setting (outcomes)  2.6 Biofeedback  3.1 Social support (unspecified)  4.4 Behavioural experiments  5.1 Information about health consequences  7.1 Prompts / cues  7.3 Reduce prompts / cues  8.2 Behavioural substitution  9.1 Credible source |
|  |  | Activity | 1.1 Goal setting (behaviour)  1.2 Problem solving  2.3 Self-monitoring of behaviour  2.6 Biofeedback  3.1 Social support (unspecified)  4.4 Behavioural experiments  7.1 Prompts / cues  7.3 Reduce prompts / cues  9.1 Credible source |
| Furler, 2020 | - Blinded CGM - Discussion of CGM results with a health care provider | CGM | 2.6 Biofeedback  3.1 Social support (unspecified)  7.1 Prompts / cues  9.1 Credible source |
|  |  | Diet | 3.1 Social support (unspecified)  7.1 Prompts / cues  9.1 Credible source |
|  |  | Activity | 3.1 Social support (unspecified)  7.1 Prompts / cues  9.1 Credible source |
| Guo, 2023 | - Unblinded CGM - Mobile app with guidance from general practitioners - Diet tracking - Activity tracking - Weight tracking - Personalized diet, exercise, and health education program based on participant’s health data | CGM | 1.6 Biofeedback  3.1 Social support (unspecified)  4.2 Information about antecedents  5.1 Information about health consequences  7.1 Prompts / cues  9.1 Credible source |
|  |  | Diet | 1.1 Goal setting (behaviour)  1.4 Action planning  2.3 Self-monitoring of behaviour  2.4 Self-monitoring of outcome(s) of behaviour  2.6 Biofeedback  3.1 Social support (unspecified)  4.2 Information about antecedents  5.1 Information about health consequences  7.1 Prompts / cues  9.1 Credible source |
|  |  | Activity | 1.1 Goal setting (behaviour)  1.4 Action planning  2.4 Self-monitoring of outcome(s) of behaviour  3.1 Social support (unspecified)  4.2 Information about antecedents  5.1 Information about health consequences  5.4 Monitoring of emotional consequences  7.1 Prompts / cues  9.1 Credible source |
| Haak, 2017 | - Unblinded CGM - Discussion of CGM results with a health care provider in relation to diet, lifestyle, and medication dosage | CGM | 2.6 Biofeedback  3.1 Social support (unspecified) |
|  |  | Diet | 2.6 Biofeedback  3.1 Social support (unspecified) |
| Jospe, 2020 | - Unblinded CGM - Education on meal timing based on glucose levels - Diet tracking | CGM | 1.4 Action planning  2.4 Self-monitoring of outcome(s) of behaviour  2.6 Biofeedback  4.1 Instruction on how to perform the behaviour  4.2 Information about antecedents  4.4 Behavioural experiments  5.1 Information about health consequences  7.1 Prompts / cues |
|  |  | Diet | 1.4 Action planning  2.3 Self-monitoring of behaviour  2.4 Self-monitoring of outcome(s) of behaviour  2.6 Biofeedback  4.1 Instruction on how to perform the behaviour  4.2 Information about antecedents  4.4 Behavioural experiments  5.1 Information about health consequences  5.4 Monitoring of emotional consequences  7.1 Prompts / cues  12.4 Distraction |
| Lee, 2022 (Intervention #1) | - Unblinded CGM - Education based on CGM data regarding diet, activity, and medication dosage | CGM | 2.6 Biofeedback  3.1 Social support (unspecified)  4.1 Instruction on how to perform the behaviour  5.1 Information about health consequences  7.1 Prompts / cues |
|  |  | Diet | 2.6 Biofeedback  3.1 Social support (unspecified)  4.1 Instruction on how to perform the behaviour  5.1 Information about health consequences |
|  |  | Activity | 2.6 Biofeedback  3.1 Social support (unspecified)  5.1 Information about health consequences |
|  |  | Medication adherence | 4.1 Instruction on how to perform the behaviour |
| Lee, 2022 (Intervention #2) | - Unblinded CGM | CGM | 2.6 Biofeedback  7.1 Prompts / cues |
| Lee, 2023 | - Unblinded CGM - Digital integrated health care platform - Feedback from medical staff on health data - Glucometer - Sphygmomanometer - Body weight scale - Pedometer - Diet tracking | CGM | 1.6 Discrepancy between current behaviour and goal  2.6 Biofeedback  2.7 Feedback on outcome(s) of behaviour  3.1 Social support (unspecified)  5.1 Information about health consequences  9.1 Credible source  10.3 Non-specific reward |
|  |  | Diet | 1.6 Discrepancy between current behaviour and goal  2.2 Feedback on behaviour  2.6 Biofeedback  2.7 Feedback on outcome(s) of behaviour  3.1 Social support (unspecified)  4.1 Instruction on how to perform the behaviour  5.1 Information about health consequences  9.1 Credible source  10.3 Non-specific reward |
|  |  | Activity | 1.6 Discrepancy between current behaviour and goal  2.2 Feedback on behaviour  2.7 Feedback on outcome(s) of behaviour  3.1 Social support (unspecified)  5.1 Information about health consequences  9.1 Credible source  10.3 Non-specific reward |
| Meisenhelder-Smith, 2006 | - Unblinded CGM - Diabetes self-management education - Diet tracking - Activity tracking - Medication tracking - Glucose tracking - Discussion of CGM results with a health care provider | CGM | 1.1 Goal setting (behaviour)  1.2 Problem solving  1.3 Goal setting (outcome)  1.5 Review behaviour goals  1.8 Behavioural contract  2.2 Feedback on behaviour  2.4 Self-monitoring of outcome(s) of behaviour  2.6 Biofeedback  3.2 Social support (practical)  3.3 Social support (emotional)  4.1 Instruction on how to perform the behaviour  5.1 Information about health consequences  5.3 Information about social and environmental consequences  5.6 Information about emotional consequences  6.1 Demonstration of the behaviour  8.1 Behavioural practice / rehearsal  9.1 Credible source |
|  |  | Diet | 1.1 Goal setting (behaviour)  1.2 Problem solving  1.3 Goal setting (outcome)  1.5 Review behaviour goals  1.8 Behavioural contract  2.2 Feedback on behaviour  2.3 Self-monitoring of behaviour  3.2 Social support (practical)  3.3 Social support (emotional)  4.1 Instruction on how to perform the behaviour  5.1 Information about health consequences  5.3 Information about social and environmental consequences  5.6 Information about emotional consequences  6.1 Demonstration of the behaviour  8.1 Behavioural practice / rehearsal  9.1 Credible source  10.7 Self-incentive  10.9 Self-reward |
|  |  | Activity | 1.1 Goal setting (behaviour)  1.2 Problem solving  1.3 Goal setting (outcome)  1.5 Review behaviour goals  1.8 Behavioural contract  2.2 Feedback on behaviour  2.3 Self-monitoring of behaviour  3.2 Social support (practical)  3.3 Social support (emotional)  4.1 Instruction on how to perform the behaviour  5.1 Information about health consequences  5.3 Information about social and environmental consequences  5.6 Information about emotional consequences  6.1 Demonstration of the behaviour  8.1 Behavioural practice / rehearsal  9.1 Credible source  10.7 Self-incentive  10.9 Self-reward |
|  |  | Medication adherence | 1.1 Goal setting (behaviour)  1.2 Problem solving  1.3 Goal setting (outcome)  1.5 Review behaviour goals  1.8 Behavioural contract  2.2 Feedback on behaviour  3.2 Social support (practical)  3.3 Social support (emotional)  4.1 Instruction on how to perform the behaviour  5.1 Information about health consequences  5.3 Information about social and environmental consequences  5.6 Information about emotional consequences  6.1 Demonstration of the behaviour  8.1 Behavioural practice / rehearsal  9.1 Credible source  10.7 Self-incentive  10.9 Self-reward |
| Murphy, 2008 | - Blinded CGM - Discussion of CGM results with a health care provider in relation to diet, activity, and medication - Antenatal care | CGM | 1.2 Problem solving  1.3 Goal setting (outcome)  1.6 Discrepancy between current behaviour and goal  2.6 Biofeedback  3.1 Social support (unspecified)  4.1 Instruction on how to perform the behaviour  4.2 Information about antecedents  9.1 Credible source  15.3 Focus on past success |
|  |  | Diet | 1.2 Problem solving  1.3 Goal setting (outcome)  2.6 Biofeedback  3.1 Social support (unspecified)  4.2 Information about antecedents  9.1 Credible source |
|  |  | Activity | 1.2 Problem solving  2.6 Biofeedback  3.1 Social support (unspecified)  4.2 Information about antecedents  9.1 Credible source |
| Price, 2021 | - Unblinded CGM - CGM-related learning modules - Discussion about CGM results with healthcare provider | CGM | 2.4 Self-monitoring of outcome(s) of behaviour  2.6 Biofeedback  3.2 Social support (practical)  4.1 Instruction on how to perform the behaviour  4.2 Information about antecedents  4.4 Behavioural experiments  9.1 Credible source |
|  |  | Diet | 2.3 Self-monitoring of behaviour  2.6 Biofeedback  4.1 Instruction on how to perform the behaviour  4.2 Information about antecedents  4.4 Behavioural experiments  5.1 Information about health consequences |
| Ruissen, 2023 | - Unblinded CGM - Glucometer - Wearable activity tracker - Web-based shared decision-making tool - App- or web-based self-management support system - Diet tracking - Activity tracking - Glucose tracking | CGM | 1.1 Goal setting (behaviour)  1.2 Problem solving  1.3 Goal setting (outcome)  1.5 Review behaviour goals  1.6 Discrepancy between current behaviour and goal  2.2 Feedback on behaviour  2.6 Biofeedback  3.2 Social support (practical)  3.3 Social support (emotional)  4.1 Instruction on how to perform the behaviour  4.2 Information about antecedents  5.1 Information about health consequences  5.3 Information about social and environmental consequences  5.4 Monitoring of emotional consequences  8.7 Graded tasks  9.1 Credible source  11.2 Reduce negative emotions  13.4 Valued self-identity  15.4 Focus on past success |
|  |  | Glucometer | 1.1 Goal setting (behaviour)  1.2 Problem solving  1.3 Goal setting (outcome)  1.5 Review behaviour goals  1.6 Discrepancy between current behaviour and goal  2.2 Feedback on behaviour  2.6 Biofeedback  3.2 Social support (practical)  3.3 Social support (emotional)  4.1 Instruction on how to perform the behaviour  4.2 Information about antecedents  5.1 Information about health consequences  5.3 Information about social and environmental consequences  5.4 Monitoring of emotional consequences  8.7 Graded tasks  9.1 Credible source  11.2 Reduce negative emotions  13.4 Valued self-identity  15.4 Focus on past success |
|  |  | Diet | 1.1 Goal setting (behaviour)  1.2 Problem solving  1.3 Goal setting (outcome)  1.5 Review behaviour goals  1.6 Discrepancy between current behaviour and goal  2.2 Feedback on behaviour  2.3 Self-monitoring of behaviour  2.4 Self-monitoring of outcome(s) of behaviour  3.2 Social support (practical)  3.3 Social support (emotional)  4.1 Instruction on how to perform the behaviour  4.2 Information about antecedents  5.1 Information about health consequences  5.3 Information about social and environmental consequences  5.4 Monitoring of emotional consequences  8.7 Graded tasks  9.1 Credible source  11.2 Reduce negative emotions  13.4 Valued self-identity  15.4 Focus on past success |
|  |  | Activity | 1.1 Goal setting (behaviour)  1.2 Problem solving  1.3 Goal setting (outcome)  1.5 Review behaviour goals  1.6 Discrepancy between current behaviour and goal  2.2 Feedback on behaviour  2.3 Self-monitoring of behaviour  2.4 Self-monitoring of outcome(s) of behaviour  3.2 Social support (practical)  3.3 Social support (emotional)  4.1 Instruction on how to perform the behaviour  4.2 Information about antecedents  5.1 Information about health consequences  5.3 Information about social and environmental consequences  5.4 Monitoring of emotional consequences  8.7 Graded tasks  9.1 Credible source  11.2 Reduce negative emotions  13.4 Valued self-identity  15.4 Focus on past success |
|  |  | Medication adherence | 1.2 Problem solving  1.5 Review behaviour goals  1.6 Discrepancy between current behaviour and goal  2.2 Feedback on behaviour  3.2 Social support (practical)  3.3 Social support (emotional)  4.1 Instruction on how to perform the behaviour  4.2 Information about antecedents  5.1 Information about health consequences  5.3 Information about social and environmental consequences  5.4 Monitoring of emotional consequences  8.7 Graded tasks  11.2 Reduce negative emotions  13.4 Valued self-identity  15.4 Focus on past success |
| Sato, 2016 | - Blinded CGM - Treatment guidance based on CGM data - Diet tracking | CGM | 2.6 Biofeedback |
|  |  | Diet | 1.2 Problem solving  2.3 Self-monitoring of behaviour  4.1 Instruction on how to perform the behaviour  9.1 Credible source |
| Schembre, 2022 | - Unblinded CGM - Education on meal timing based on glucose levels - Diabetes Prevention Program - Weight tracking - Group exercise classes | CGM | 2.4 Self-monitoring of outcome(s) of behaviour  2.6 Biofeedback  3.1 Social support (unspecified)  4.1 Instruction on how to perform the behaviour |
|  |  | Diet | 1.1 Goal setting (behaviour)  1.2 Problem solving  1.3 Goal setting (outcome)  1.4 Action planning  2.4 Self-monitoring of outcome(s) of behaviour  2.6 Biofeedback  3.1 Social support (unspecified)  4.1 Instruction on how to perform the behaviour  5.3 Information about social and environmental consequences  9.1 Credible source  11.2 Reduce negative emotions  12.3 Avoidance / reducing exposure to cues for the behaviour  15.4 Self-talk |
|  |  | Activity | 1.1 Goal setting (behaviour)  1.2 Problem solving  1.3 Goal setting (outcome)  2.4 Self-monitoring of outcome(s) of behaviour  4.1 Instruction on how to perform the behaviour  5.3 Information about social and environmental consequences  6.1 Demonstration of the behaviour  8.1 Behavioural practice / rehearsal  9.1 Credible source  11.2 Reduce negative emotions  12.3 Avoidance / reducing exposure to cues for the behaviour  15.4 Self-talk |
| Taylor, 2019 | - Unblinded CGM - Glucometer - Glucose tracking - Diet tracking - Activity tracking - Nutrition education - Low-carbohydrate, high-protein and unsaturated fat diet - Exercise plan - Discussion of CGM data with healthcare provider | CGM | 2.6 Biofeedback  3.2 Social support (practical) |
|  |  | Glucometer | 2.4 Self-monitoring of outcome(s) of behaviour  2.6 Biofeedback  4.1 Instruction on how to perform the behaviour  8.2 Behavioural substitution |
|  |  | Diet | 1.3 Goal setting (outcome)  1.4 Action planning  2.6 Biofeedback  4.1 Instruction on how to perform the behaviour |
|  |  | Activity | 4.1 Instruction on how to perform the behaviour |
| Tumminia, 2021 (Intervention #1) | - Unblinded CGM - Glucometer - Structured diabetes education program - Treatment adjustments based on CGM data | CGM | 2.6 Biofeedback  3.1 Social support (unspecified)  4.1 Instruction on how to perform the behaviour  5.1 Information about health consequences  9.1 Credible source |
|  |  | Glucometer | 2.6 Biofeedback  3.1 Social support (unspecified)  5.1 Information about health consequences |
|  |  | Diet | 2.6 Biofeedback  3.1 Social support (unspecified)  4.1 Instruction on how to perform the behaviour  5.1 Information about health consequences  9.1 Credible source |
|  |  | Activity | 2.6 Biofeedback  3.1 Social support (unspecified)  4.1 Instruction on how to perform the behaviour  5.1 Information about health consequences  9.1 Credible source |
| Tumminia, 2021 (Intervention #2) | - Unblinded CGM - Structured diabetes education program - Treatment adjustments based on glucometer data | CGM | 2.6 Biofeedback  3.1 Social support (unspecified)  4.1 Instruction on how to perform the behaviour  5.1 Information about health consequences  9.1 Credible source |
|  |  | Diet | 2.6 Biofeedback  3.1 Social support (unspecified)  4.1 Instruction on how to perform the behaviour  5.1 Information about health consequences  9.1 Credible source |
|  |  | Activity | 2.6 Biofeedback  3.1 Social support (unspecified)  4.1 Instruction on how to perform the behaviour  5.1 Information about health consequences  9.1 Credible source |
| Voormolen, 2018 | - Blinded CGM - Glucometer - Discussion of CGM results with health care professional in relation to diet and medication | CGM | 2.6 Biofeedback |
|  |  | Glucometer | 1.3 Goal setting (outcome)  2.4 Self-monitoring of outcome(s) of behaviour  2.6 Biofeedback  3.1 Social support (unspecified)  4.1 Instruction on how to perform the behaviour |
|  |  | Diet | 2.3 Self-monitoring of behaviour  2.6 Biofeedback  3.1 Social support (unspecified) |
|  |  | Activity | 2.3 Self-monitoring of behaviour  2.6 Biofeedback  3.1 Social support (unspecified) |
| Wada, 2020 | - Unblinded CGM - Education on how to adjust behaviours based on glucose levels | CGM | 1.3 Goal setting (outcome)  2.6 Biofeedback  4.1 Instruction on how to perform the behaviour |
|  |  | Diet | 2.6 Biofeedback  4.1 Instruction on how to perform the behaviour |
|  |  | Activity | 4.1 Instruction on how to perform the behaviour |
| Yan, 2022 | - Unblinded CGM - Diet tracking - Activity tracking - Treatment adjustment based on CGM data - Diabetes education | CGM | 2.6 Biofeedback  4.1 Instruction on how to perform the behaviour |
|  |  | Glucometer | 2.6 Biofeedback |
|  |  | Diet | 2.3 Self-monitoring of behaviour  2.6 Biofeedback  4.1 Instruction on how to perform the behaviour |
|  |  | Activity | 2.3 Self-monitoring of behaviour  2.6 Biofeedback  4.1 Instruction on how to perform the behaviour |
| Yeoh, 2018 | - Blinded CGM - Glucometer - Diet tracking - Activity tracking - Treatment adjustment based on CGM data in relation to medication and lifestyle | CGM | 2.6 Biofeedback  3.1 Social support (unspecified) |
|  |  | Glucometer | 2.6 Biofeedback  4.1 Instruction on how to perform the behaviour |
|  |  | Diet | 2.3 Self-monitoring of behaviour  2.6 Biofeedback  3.1 Social support (unspecified)  4.1 Instruction on how to perform the behaviour |
|  |  | Activity | 2.3 Self-monitoring of behaviour  2.6 Biofeedback  3.1 Social support (unspecified)  4.1 Instruction on how to perform the behaviour |
| Yoo, 2008 | - Unblinded CGM - Guidance to increase activity and reduce food intake when hyperglycaemic alarms occur - Discussion of CGM results with a health care provider in relation to diet and activity | CGM | 2.6 Biofeedback |
|  |  | Glucometer | 1.4 Action planning  7.1 Prompts / cues |
|  |  | Diet | 1.4 Action planning  2.6 Biofeedback  7.1 Prompts / cues  9.1 Credible source |
|  |  | Activity | 1.4 Action planning  2.6 Biofeedback  7.1 Prompts / cues  9.1 Credible source |
| W. Zhang, 2021 | - Unblinded CGM - Glucometer - Treatment adjustments based on CGM data in relation to diet and medication | CGM | 2.6 Biofeedback  4.1 Instruction on how to perform the behaviour |
|  |  | Glucometer | 2.6 Biofeedback |
|  |  | Diet | 4.1 Instruction on how to perform the behaviour |
| X. Zhang, 2021 | - Unblinded CGM - Glucometer - Discussion of CGM data with a health care provider, with treatment adjustments as necessary - Home diet and activity plans based on glucose data - Diet tracking - Activity tracking - Medication tracking - Hypoglycaemic event tracking | CGM | 2.4 Self-monitoring of outcome(s) of behaviour  2.6 Biofeedback |
|  |  | Glucometer | 2.4 Self-monitoring of outcome(s) of behaviour  2.6 Biofeedback |
|  |  | Diet | 2.3 Self-monitoring of behaviour  2.4 Self-monitoring of outcome(s) of behaviour  2.6 Biofeedback  4.1 Instruction on how to perform the behaviour |
|  |  | Activity | 2.3 Self-monitoring of behaviour  2.4 Self-monitoring of outcome(s) of behaviour  2.6 Biofeedback  4.1 Instruction on how to perform the behaviour |
| **Control arms** | | | |
| Alfadhli, 2016 | - Glucometer | Glucometer | 1.3 Goal setting (outcome)  2.3 Self-monitoring of behaviour  2.4 Self-monitoring of outcome(s)  2.6 Biofeedback |
|  |  | Diet | 1.3 Goal setting (outcome)  2.3 Self-monitoring of behaviour  2.4 Self-monitoring of outcome(s) |
| Allen, 2008 | - Diabetes education | Glucometer | 3.2 Social support (practical)  4.1 Instruction on how to perform the behaviour |
|  |  | Diet | 1.1 Goal setting (behaviour)  1.2 Problem solving  3.2 Social support (practical)  4.1 Instruction on how to perform the behaviour |
|  |  | Activity | 1.1 Goal setting (behaviour)  1.2 Problem solving  3.2 Social support (practical)  4.1 Instruction on how to perform the behaviour |
| Aronson, 2023 | - Glucometer - Diabetes self-management education | Glucometer | 1.1 Goal setting (behaviour)  1.5 Review behaviour goals  2.3 Self-monitoring of behaviour  2.4 Self-monitoring of outcome(s) of behaviour  2.6 Biofeedback  3.1 Social support (unspecified)  4.1 Instruction on how to perform the behaviour  4.4 Behavioural experiments  5.1 Information about health consequences  5.4 Monitoring of emotional consequences  7.1 Prompts/cues  9.1 Credible source |
|  |  | Diet | 1.1 Goal setting (behaviour)  1.5 Review behaviour goals  2.3 Self-monitoring of behaviour  2.4 Self-monitoring of outcome(s) of behaviour  2.6 Biofeedback  3.1 Social support (unspecified)  4.1 Instruction on how to perform the behaviour  4.4 Behavioural experiments  5.1 Information about health consequences  5.4 Monitoring of emotional consequences  9.1 Credible source |
|  |  | Activity | 1.1 Goal setting (behaviour)  1.5 Review behaviour goals  2.3 Self-monitoring of behaviour  2.4 Self-monitoring of outcome(s) of behaviour  2.6 Biofeedback  3.1 Social support (unspecified)  4.1 Instruction on how to perform the behaviour  4.4 Behavioural experiments  5.1 Information about health consequences  5.4 Monitoring of emotional consequences  9.1 Credible source |
|  |  | Medication adherence | 1.1 Goal setting (behaviour)  1.5 Review behaviour goals  2.3 Self-monitoring of behaviour  2.4 Self-monitoring of outcome(s) of behaviour  2.6 Biofeedback  3.1 Social support (unspecified)  4.1 Instruction on how to perform the behaviour  4.4 Behavioural experiments  5.1 Information about health consequences  5.4 Monitoring of emotional consequences  9.1 Credible source |
| Chekima, 2022 | - Nutrition education on low glycaemic index and glycaemic load foods | Diet | 4.1 Instruction on how to perform the behaviour |
|  |  |  |  |
| Choe, 2022 | - Glucometer - Glucose tracking | Glucometer | 2.4 Self-monitoring of outcome(s) of behaviour  2.6 Biofeedback |
|  |  | Diet | 2.4 Self-monitoring of outcome(s) of behaviour  2.6 Biofeedback |
| Cosson, 2009 | - Blinded CGM - Glucometer - Treatment adjustments based on glucometer data | CGM | 2.5 Monitoring of outcome(s) of behaviour without feedback |
|  |  | Glucometer | 2.6 Biofeedback  3.2 Social support (practical)  4.1 Instruction on how to perform the behaviour  5.1 Information about health consequences |
|  |  | Diet | 3.2 Social support (practical)  4.1 Instruction on how to perform the behaviour  5.1 Information about health consequences |
|  |  | Activity | 3.2 Social support (practical)  4.1 Instruction on how to perform the behaviour  5.1 Information about health consequences |
| Cox, 2020 (Control #1) | - Education on nutrition and weight loss - Diet tracking - Activity tracking | Diet | 1.1 Goal setting (behaviour)  1.2 Problem solving  2.2 Feedback on behaviour  2.3 Self-monitoring of behaviour  3.1 Social support (unspecified)  4.1 Instruction on how to perform the behaviour  9.1 Credible source  11.2 Reduce negative emotions |
|  |  | Activity | 1.1 Goal setting (behaviour)  1.2 Problem solving  2.2 Feedback on behaviour  2.3 Self-monitoring of behaviour  3.1 Social support (unspecified)  4.1 Instruction on how to perform the behaviour  9.1 Credible source  11.2 Reduce negative emotions |
| Cox, 2020 (Control #2) | - Education on low-glycaemic load foods, increasing physical activity, and decreasing sedentary time - Diet tracking - Activity tracking | Diet | 1.2 Problem solving  1.3 Goal setting (outcome)  3.1 Social support (unspecified)  8.2 Behavioural Substitution  9.1 Credible source |
|  |  | Activity | 1.1 Goal setting (behaviour)  1.2 Problem solving  2.3 Self-monitoring of behaviour  3.1 Social support (unspecified)  9.1 Credible source |
| Cox, 2020 (Control #3) | - Glucometer - Education on low-glycaemic load foods, increasing physical activity, and decreasing sedentary time - Diet tracking - Activity tracking - Glucose tracking | Glucometer | 2.6 Biofeedback  3.1 Social support (unspecified)  4.1 Instruction on how to perform the behaviour  4.4 Behavioural experiments  5.1 Information about health consequences  7.3 Reduce prompts / cues  9.1 Credible source |
|  |  | Diet | 1.2 Problem solving  1.3 Goal setting (outcome)  2.6 Biofeedback  3.1 Social support (unspecified)  4.4 Behavioural experiments  5.1 Information about health consequences  7.3 Reduce Prompts Cues  8.2 Behavioural Substitution  9.1 Credible source |
|  |  | Activity | 1.1 Goal setting (behaviour)  1.2 Problem solving  2.3 Self-monitoring of behaviour  2.6 Biofeedback  3.1 Social support (unspecified)  4.4 Behavioural experiments  7.3 Reduce prompts / cues  9.1 Credible source |
| Furler, 2020 | - Blinded CGM - Usual care | CGM | 2.5 Monitoring of outcome(s) of behaviour without feedback |
|  |  | Diet | 3.1 Social support (unspecified)  9.1 Credible source |
|  |  | Activity | 3.1 Social support (unspecified)  9.1 Credible source |
| Guo, 2023 | - Glucometer - Education on type 2 diabetes, diet, exercise, and diabetes prevention | Glucometer | 2.6 Biofeedback  3.1 Social support (unspecified)  9.1 Credible source |
|  |  | Diet | 4.1 Instruction on how to perform the behaviour  5.1 Information about health consequences |
|  |  | Activity | 4.1 Instruction on how to perform the behaviour  5.1 Information about health consequences |
| Haak, 2017 | - Glucometer - Glucose tracking - Discussion of glucometer data with a health care provider in relation to diet, lifestyle, and medication dosage | Glucometer | 2.4 Self-monitoring of outcome(s) of behaviour  2.6 Biofeedback  3.1 Social support (unspecified) |
|  |  | Diet | 2.6 Biofeedback  3.1 Social support (unspecified) |
| Jospe, 2020 | - Glucometer - Education on meal timing based on glucose levels - Diet tracking | Glucometer | 1.4 Action planning  2.4 Self-monitoring of outcome(s) of behaviour  2.6 Biofeedback  4.1 Instruction on how to perform the behaviour  4.2 Information about antecedents  4.4 Behavioural experiments  5.1 Information about health consequences  7.1 Prompts/cues |
|  |  | Diet | 1.4 Action planning  2.3 Self-monitoring of behaviour  2.4 Self-monitoring of outcome(s) of behaviour  2.6 Biofeedback  4.1 Instruction on how to perform the behaviour  4.2 Information about antecedents  4.4 Behavioural experiments  5.1 Information about health consequences  5.4 Monitoring of emotional consequences  7.1 Prompts/cues  12.4 Distraction |
| Lee, 2023 (Control #1) | - Routine diabetes care | *N/A* | *N/A* |
| Lee, 2023 (Control #2) | - Digital integrated health care platform - Glucometer - Sphygmomanometer - Body weight scale - Pedometer - Diet tracking | Glucometer | 2.6 Biofeedback |
|  |  | Diet | 2.3 Self-monitoring of behaviour  2.4 Self-monitoring of outcome(s) of behaviour |
|  |  | Activity | 2.3 Self-monitoring of behaviour  2.4 Self-monitoring of outcome(s) of behaviour |
| Meisenhelder-Smith, 2006 | - Glucometer - Diabetes self-management education | Glucometer | 1.1 Goal setting (behaviour)  1.2 Problem solving  1.3 Goal setting (outcome)  1.5 Review behaviour goals  1.8 Behavioural contract  2.2 Feedback on behaviour  2.4 Self-monitoring of outcome(s) of behaviour  2.6 Biofeedback  3.2 Social support (practical)  3.3 Social support (emotional)  4.1 Instruction on how to perform the behaviour  5.1 Information about health consequences  5.3 Information about social and environmental consequences  5.6 Information about emotional consequences  6.1 Demonstration of the behaviour  8.1 Behavioural practice/rehearsal  9.1 Credible source |
|  |  | Diet | 1.1 Goal setting (behaviour)  1.2 Problem solving  1.3 Goal setting (outcome)  1.5 Review behaviour goals  1.8 Behavioural contract  10.7 Self-incentive  10.9 Self-reward  2.2 Feedback on behaviour  2.3 Self-monitoring of behaviour  3.2 Social support (practical)  3.3 Social support (emotional)  4.1 Instruction on how to perform the behaviour  5.1 Information about health consequences  5.3 Information about social and environmental consequences  5.6 Information about emotional consequences  6.1 Demonstration of the behaviour  8.1 Behavioural practice/rehearsal  9.1 Credible source |
|  |  | Activity | 1.1 Goal setting (behaviour)  1.2 Problem solving  1.3 Goal setting (outcome)  1.5 Review behaviour goals  1.8 Behavioural contract  10.7 Self-incentive  10.9 Self-reward  2.2 Feedback on behaviour  2.3 Self-monitoring of behaviour  3.2 Social support (practical)  3.3 Social support (emotional)  4.1 Instruction on how to perform the behaviour  5.1 Information about health consequences  5.3 Information about social and environmental consequences  5.6 Information about emotional consequences  6.1 Demonstration of the behaviour  8.1 Behavioural practice/rehearsal  9.1 Credible source |
|  |  | Medication adherence | 1.1 Goal setting (behaviour)  1.2 Problem solving  1.3 Goal setting (outcome)  1.5 Review behaviour goals  1.8 Behavioural contract  10.7 Self-incentive  10.9 Self-reward  2.2 Feedback on behaviour  3.2 Social support (practical)  3.3 Social support (emotional)  4.1 Instruction on how to perform the behaviour  5.1 Information about health consequences  5.3 Information about social and environmental consequences  5.6 Information about emotional consequences  6.1 Demonstration of the behaviour  8.1 Behavioural practice/rehearsal  9.1 Credible source |
| Murphy, 2008 | - Standard antenatal care | Glucometer | 1.3 Goal setting (outcome)  2.6 Biofeedback  3.1 Social support (unspecified)  4.1 Instruction on how to perform the behaviour  9.1 Credible source |
|  |  | Diet | 3.1 Social support (unspecified)  9.1 Credible source |
|  |  | Activity | 1.3 Goal setting (outcome)  3.1 Social support (unspecified)  9.1 Credible source |
| Price, 2021 | - Glucometer - Discussion about glucometer data with healthcare provider | Glucometer | 2.6 Biofeedback  3.2 Social support (practical)  9.1 Credible source |
| Ruissen, 2023 | - Usual care | Glucometer | 3.1 Social support (unspecified)  9.1 Credible source |
| Sato, 2016 | - Blinded CGM - Glucometer - Treatment guidance based on glucometer data - Diet tracking | CGM | 2.5 Monitoring of outcome(s) of behaviour |
|  |  | Glucometer | 2.6 Biofeedback |
|  |  | Diet | 2.3 Self-monitoring of behaviour |
| Schembre, 2022 | - Diabetes Prevention Program - Weight tracking - Group exercise classes | Diet | 1.1 Goal setting (behaviour)  1.2 Problem solving  1.3 Goal setting (outcome)  2.4 Self-monitoring of outcome(s) of behaviour  4.1 Instruction on how to perform the behaviour  5.3 Information about social and environmental consequences  9.1 Credible source  11.2 Reduce negative emotions  12.3 Avoidance/reducing exposure to cues for the behaviour  15.4 Self-talk |
|  |  | Activity | 1.1 Goal setting (behaviour)  1.2 Problem solving  1.3 Goal setting (outcome)  2.4 Self-monitoring of outcome(s) of behaviour  4.1 Instruction on how to perform the behaviour  5.3 Information about social and environmental consequences  6.1 Demonstration of the behaviour  8.1 Behavioural practice/rehearsal  9.1 Credible source  11.2 Reduce negative emotions  12.3 Avoidance/reducing exposure to cues for the behaviour  15.4 Self-talk |
| Taylor, 2019 | - Blinded CGM - Glucometer - Glucose tracking - Diet tracking - Activity tracking - Nutrition education - Low-carbohydrate, high-protein and unsaturated fat diet - Exercise plan | Glucometer | 2.4 Self-monitoring of outcome(s) of behaviour  2.6 Biofeedback  4.1 Instruction on how to perform the behaviour |
|  |  | Diet | 1.3 Goal setting (outcome)  1.4 Action planning  2.6 Biofeedback  4.1 Instruction on how to perform the behaviour  8.2 Behavioural Substitution |
|  |  | Activity | 4.1 Instruction on how to perform the behaviour |
| Voormolen, 2018 | - Glucometer | Glucometer | 1.3 Goal setting (outcome)  2.4 Self-monitoring of outcome(s) of behaviour  2.6 Biofeedback  3.1 Social support (unspecified)  4.1 Instruction on how to perform the behaviour |
|  |  | Diet | 2.3 Self-monitoring of behaviour  2.6 Biofeedback  3.1 Social support (unspecified) |
|  |  | Activity | 2.3 Self-monitoring of behaviour  2.6 Biofeedback  3.1 Social support (unspecified) |
| Wada, 2020 | - Blinded CGM - Glucometer - Education on how to adjust behaviours based on glucose levels | CGM | 2.5 Monitoring of outcome(s) of behaviour |
|  |  | Glucometer | 1.3 Goal setting (outcome)  2.6 Biofeedback  4.1 Instruction on how to perform the behaviour |
|  |  | Diet | 2.6 Biofeedback  4.1 Instruction on how to perform the behaviour |
|  |  | Activity | 4.1 Instruction on how to perform the behaviour |
| Yan, 2022 | - Blinded CGM - Diet tracking - Activity tracking - Treatment adjustment based on CGM data regarding medications - Diabetes education | CGM | 2.5 Monitoring of outcome(s) of behaviour without feedback |
|  |  | Glucometer | 2.6 Biofeedback |
|  |  | Diet | 2.3 Self-monitoring of behaviour  2.6 Biofeedback |
|  |  | Activity | 2.3 Self-monitoring of behaviour  2.6 Biofeedback |
| Yeoh, 2018 | - Glucometer - Glucose tracking - Review of glucose data with trial investigators | Glucometer | 2.6 Biofeedback  4.1 Instruction on how to perform the behaviour |
|  |  | Diet | 2.6 Biofeedback  4.1 Instruction on how to perform the behaviour |
|  |  | Activity | 2.6 Biofeedback  4.1 Instruction on how to perform the behaviour |
| Yoo, 2008 | - Glucometer - Standard diabetes education - Discussion of glucometer results with a health care provider regarding diet and activity | Glucometer | 2.6 Biofeedback  4.1 Instruction on how to perform the behaviour |
|  |  | Diet | 2.6 Biofeedback  2.7 Feedback on outcome(s) of behaviour  4.1 Instruction on how to perform the behaviour |
|  |  | Activity | 2.6 Biofeedback  2.7 Feedback on outcome(s) of behaviour  4.1 Instruction on how to perform the behaviour |
| W. Zhang, 2021 | - Glucometer - Treatment adjustments based on glucometer data regarding diet and medication | CGM | 2.5 Monitoring of outcome(s) of behaviour without feedback |
|  |  | Glucometer | 2.6 Biofeedback |
|  |  | Diet | 4.1 Instruction on how to perform the behaviour |
| X. Zhang, 2021 | - Glucometer - Home diet and activity plans based on glucose data | Glucometer | 2.4 Self-monitoring of outcome(s) of behaviour  2.6 Biofeedback |
|  |  | Diet | 2.4 Self-monitoring of outcome(s) of behaviour  2.6 Biofeedback |
|  |  | Activity | 2.4 Self-monitoring of outcome(s) of behaviour  2.6 Biofeedback |
